# Chemical Potential Tuning and Enhancement of Thermoelectric Properties in Indium Selenides

**DOI:** 10.3390/ma8031283

**Published:** 2015-03-20

**Authors:** Jong-Soo Rhyee, Jin Hee Kim

**Affiliations:** Department of Applied Physics, Kyung Hee University, Yong-In 446-701, Korea; E-Mail: jinicool1@daum.net

**Keywords:** thermoelectric, Peierls distortion, Indium Selenides, charge density wave, chemical potential

## Abstract

Researchers have long been searching for the materials to enhance thermoelectric performance in terms of nano scale approach in order to realize phonon-glass-electron-crystal and quantum confinement effects. Peierls distortion can be a pathway to enhance thermoelectric figure-of-merit *ZT* by employing natural nano-wire-like electronic and thermal transport. The phonon-softening known as Kohn anomaly, and Peierls lattice distortion decrease phonon energy and increase phonon scattering, respectively, and, as a result, they lower thermal conductivity. The quasi-one-dimensional electrical transport from anisotropic band structure ensures high Seebeck coefficient in Indium Selenide. The routes for high *ZT* materials development of In_4_Se_3−δ_ are discussed from quasi-one-dimensional property and electronic band structure calculation to materials synthesis, crystal growth, and their thermoelectric properties investigations. The thermoelectric properties of In_4_Se_3−δ_ can be enhanced by electron doping, as suggested from the Boltzmann transport calculation. Regarding the enhancement of chemical potential, the chlorine doped In_4_Se_3−δ_Cl_0.03_ compound exhibits high *ZT* over a wide temperature range and shows state-of-the-art thermoelectric performance of *ZT* = 1.53 at 450 °C as an *n*-type material. It was proven that multiple elements doping can enhance chemical potential further. Here, we discuss the recent progress on the enhancement of thermoelectric properties in Indium Selenides by increasing chemical potential.

## 1. Introduction

The global energy crisis makes an issue for not only a development of the new eco-friendly energy sources but also the efficient consumption of the energies currently in use. Thermoelectric research is one of the efforts that promotes energy efficiency. Thermoelectric performance can be defined by the dimensionless figure-of-merit *ZT = S^2^*σ*T/*κ, where *S*, σ, κ, and *T* are Seebeck coefficient, electrical resistivity, thermal conductivity, and absolute temperature, respectively.

Recent thermoelectric research has been focused on new materials’ design as well as nano structure synthesis of the materials. The nano thermoelectric research was driven by the theoretical calculation of Hicks and Dresselhaus in 1990s. Theoretically, they argued that the quantum-well superlattice structure may have high thermoelectric performance by the low dimensional electronic transport and phonon interface scattering [1]. Experimentally, they observed the enhancement of power factor for the thickness controlled PbTe/Pb_1−*x*_Eu*_x_*Te multiple quantum well structures [2]. Since then, many attempts have been devoted to increase *ZT* in quantum well and quantum dot superstructures to enhance power factor *S^2^*σ [3,4,5]. The high *ZT* in superlattice structure accelerates the research on the nano-scale approach in thermoelectricity. Thermal conductivity can be minimized by promoting phonon localization while preserving the itineracy of the electron transport; this is called the phonon-glass and electron-crystal (PGEC) concept. The PGEC and quantum confinement in low-dimensionality become the mainstream on the research of thermoelectricity. Those concepts are based upon the low thermal conductivity by phonon scattering and increase of Seebeck coefficient by low-dimensional electronic confinement.

In spite of the reported high *ZT* value, the artificial superlattice structure is limited to practical applications for waste heat power generation because of difficulty of scaling up and maintaining temperature gradient. In order to achieve bulk scaling up, new physical concept should be employed in naturally nano-structured materials with low dimensionality of electronic transport. Several attempts were exploited to yield ultralow thermal conductivity. It was suggested that the layered structure of disordered two dimensional crystalline sheets may have extremely low thermal conductivity [6]. From this point, we proposed the possible application of Peierls distortion into the thermoelectricity.

The Peierls distortion has important ingredients to enhance thermoelectric performance. In the Peierls distortion, the charge transport is inherently low dimensional with strong electron-phonon coupling [7]. The strong electron-phonon interaction breaks the translational symmetry of lattices resulting in the lattice distortion along the transport plane. In addition, the phonon softening induced by strong electron-phonon coupling decreases the phonon energy. Firstly, the reduction of phonon energy with disordered lattices induces the significant phonon scattering. Secondly, because the Peierls distortion is a quasi-one-dimensional electronic transport phenomenon, the high Seebeck coefficient is anticipated if we control the electron-hole asymmetry [8]. Thirdly, the Peierls distortion starts from the metallic ground state, which originally describes the metal to insulator or semiconductor transition. Therefore, when we control the electron-phonon coupling via control of carrier concentration in low dimensional crystalline lattices, the energy gap can be controlled to maximize the power factor. For example, the relaxation of Te_4_ unit in Ag_10_Te_4_Br_3_ compound makes Peierls-type lattice distortion, accompanied by the delocalization of charge carriers [9]. The related materials of Ag chalcogenide ternary compounds exhibited high *ZT* with lattice distortion and crystal phase transitions [10,11,12]. Here, as a model system of Peierls distortion, we explored the In_4_Se_3_ based materials for thermoelectric properties investigations.

Even though many state-of-the-art thermoelectric materials have reportedly high *ZT* values of 2.6 at 923 K (SnSe) [13] and 2.2 at 915 K (SrTe-doped PbTe) [14], the materials with the highest record of *ZT* values are mostly focused on *p*-type materials. The *n*-type materials with high thermoelectric performance are needed as a counterpart of *p*-type in order to make thermoelectric devices. Through the quasi one-dimensional lattice distortion in In_4_Se_3−*x*_ bulk single crystals, we have achieved a high thermoelectric figure-of-merit *ZT* of 1.48 at 705 K as *n*-type materials [15]. However, two challenges still remain for practical applications. Firstly, the reported *ZT* could be enhanced further if we could increase the carrier concentration of the In_4_Se_3−*x*_ crystals because it is far from the carrier concentration of the heavily doped semiconductor (the order of 10^19^ cm^−3^) that is generally considered to be optimal for thermoelectric materials. Secondly, the high *ZT* for In_4_Se_3−*x*_ has been exhibited in bulk crystalline materials. If we synthesize high *ZT* materials as a polycrystalline compound, it has been a great importance for practical applications. The multi-elements doping can enhance chemical potential, in other words power factor, and the bulk composite can decrease thermal conductivity. Here we review the recent progress on the Indium Selenide based compounds and the efforts to increase chemical potential in order to increase *ZT* value.

## 2. Results and Discussion

### 2.1. Quasi-One-Dimensional Properties of In_4_Se_3_

Electronic band structure modification is more likely for quasi-one-dimensional materials rather than the quasi-two-dimensional ones. In order to achieve high power factor, selective charge localization of electron or hole is essential because the electron-hole mixing gives rise to Seebeck coefficient compensation. As a candidate material of quasi 1D systems, In_4_Se_3_ is very promising because it has natural nanowire-like cylindrical clusters and quasi-one-dimensional Indium chains at the cleavage (100) surface from the scanning tunneling microscopy (STM) measurements [16,17,18]. The In_4_Se_3_ has a layered crystal structure of (In_3_)^5+^ clusters covalently bonded to Se ions in the *bc*-planes held together by van der Waals interactions along the *a*-axis. The interstitial Indium resides in between the layers making relatively strong van der Waals interaction, resulting in the enhanced mechanical property.

**Figure 1:**
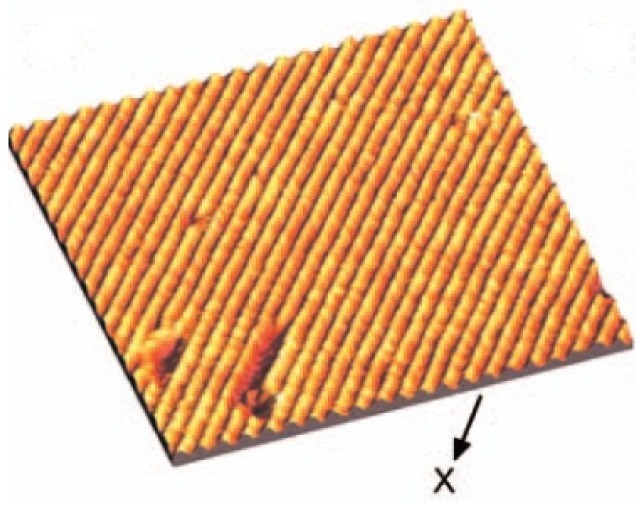
The scanning tunneling microscopy images of cleaved In_4_Se_3_. Reproduced with permission from AIP Publishing LLC, 2008 [18].

The cleavage (100) surface of In_4_Se_3_ investigated by scanning tunneling microscopy (STM) shows the quasi-one-dimensional Indium chain and nano-wire-like structure at the cleaved surface as shown in Figure 1 [16,18]. From the scanning tunneling spectroscopy (STS) and photoemission spectroscopy, the valence band maximum is located at −2 eV from the Fermi energy which is very large band gap compared to the thermoelectric materials. It was confirmed that the electronic band structure of In_4_Se_3_ is very anisotropic. The effective mass of hole along the chain direction is 5.5 times greater than the direction perpendicular to the chains, implying anisotropic band dispersion between electron and hole bands [19]. In spite of the low dimensional crystal structure and anisotropic electronic structure of In_4_Se_3_, it is known that the charge density wave is unlikely in this material [20]. However, when we tune the energy gap by controlling the carrier concentration, we can control the electron–phonon coupling so as to manifest the charge density wave.

### 2.2. Thermoelectric Properties of In_4_Se_3−δ_

#### 2.2.1. Polycrystalline Materials and Energy Band Structure

**Figure 2:**
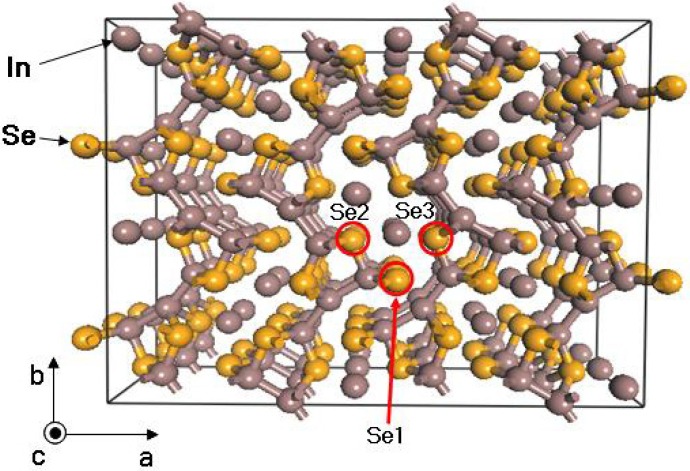
Crystal structure of In_4_Se_3_. Reproduced with permission from Nature publishing Group, 2009 [15].

In order to induce the charge density wave, we employ the Selenium deficiency. The Se deficiency has an effect on the increase of carrier concentration. From the theoretical calculation, we found that the configuration with vacant Se1 site is lower in energy than other configurations with Se2 and Se3 sites by 0.14 eV and 0.19 eV per unit cell, respectively (see Figure 2).

**Figure 3:**
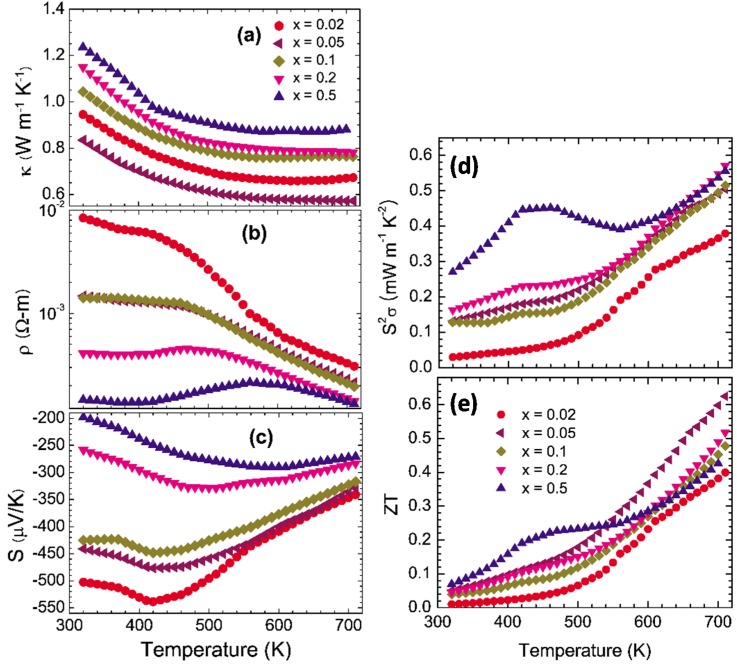
Thermoelectric properties of polycrystalline In_4_Se_3−δ_ (δ = 0.02, 0.05, 0.1, 0.2, and 0.5). (**a**) Thermal conductivity, (**b**) Electrical resistivity, (**c**) Seebeck coefficient, (**d**) Power factor, and (**e**) Dimensionless figure-of-merit. Reproduced with permission from AIP Publishing LLC, 2009 [21].

We made Se-deficient polycrystalline materials of In_4_Se_3−δ_ as shown in Figure 3 [21]. The thermal conductivity has shown that the small Se deficiency of δ = 0.05 reduces the thermal conductivity (0.83 W·m^−1^·K^−1^ at 320 K), which may arise from the random disorder phonon scattering induced by the Se deficient sites. By increasing the Se deficiency, the value of κ increases but still remains low value (1.23 W·m^−1^·K^−1^ at 320 K for *x* = 0.5). The thermal conductivity is dominated by the lattice thermal conductivity $\kappa_{ph}$ ($\kappa_{el}≅$ 0.05~0.1 W·m^−1^·K^−1^) because of the relatively high electrical resistivity of the compounds. The electrical resistivity, as shown in Figure 3b, decreases with increasing Se-deficiency *x*. The semiconducting behavior of ρ(T) was observed at high temperatures (T≥ 600 K) for the whole series of compounds. The decrease of ρ(T) with increasing Se-deficiency indicates the semi-metallic evolution of the electrical resistivity. Because of the semi-metallic variation of ρ(T), the absolute Seebeck coefficients S(T) decrease with increasing Se-deficiency, as shown in Figure 3c, from −500 μV/K (*x* = 0.02) to −198 μV/K (*x* = 0.5) at T= 320 K. The negative Seebeck coefficients indicate electronic n-type conduction by charge carriers. The power factor $S^{2}\text{σ}$ increases with increasing Se deficiency δ in the intermediate temperature range of 300 K ≤T ≤ 550 K. Owing to the low thermal conductivity and high Seebeck coefficient of the In_4_Se_3−δ_ (δ = 0.05) compound, the *ZT* reaches up to 0.63 at T = 710 K, which is comparable to the *n*-type PbTe and CoSb_3_ skutterudites in this temperature range [22].

The decrease of electrical resistivity is mainly from the increase of an effective carrier density with increase of the Se deficiency concentration δ. The effective carrier concentration $n_{\text{eff}}$, electrical resistivity ρ, Seebeck coefficient *S*, power factor $S^{2}\text{σ}$, Hall mobility of the electron $\left|{\text{μ}}_{H}\right|$, as calculated by the relationship $\left|{\text{μ}}_{H}\right|=-R_{H}(H=1\text{T})/\text{ρ}$ at T= 320 K, and effective mass of electron $m^{*}$ by following equation are summarized in Table 1:

(1) $$\text{S}=\frac{8{\text{π}}^{2}k_{B}^{2}}{3eh^{2}}m^{*}T{\left(\frac{\text{π}}{3n_{\text{eff}}}\right)}^{2/3}$$

**Table 1:** The effective carrier concentration $n_{\mathrm{eff}}$, electrical resistivity ρ, Seebeck coefficient *S*, power factor $s^{2}\text{σ}$, Hall mobility of the electron $\left|{\text{μ}}_{H}\right|$, and effective mass of electron $m^{*}$ of polycrystalline In_4_Se_3−δ_ compounds. Reproduced with permission from AIP Publishing LLC, 2009 [21].

*X*	$n_{eff}$ (10^17^ cm^−3^)	ρ (Ω·cm)	S (μV/K)	$S^{2}\sigma$ (mW m^−1^ K^−1^)	$\left|\mu_{H}\right|$ (cm^2^ V^−1^ S^−1^)	$m^{*}$ ($m_{e}$)
0.02	0.89	0.84	−502	0.030	83.4	0.047
0.05	1.74	0.15	−441	0.131	242.7	0.064
0.1	2.09	0.14	−425	0.128	212.2	0.070
0.2	12.86	0.041	−259	0.163	117.9	0.143
0.5	33.27	0.015	−198	0.270	128.8	0.206

**Figure 4:**
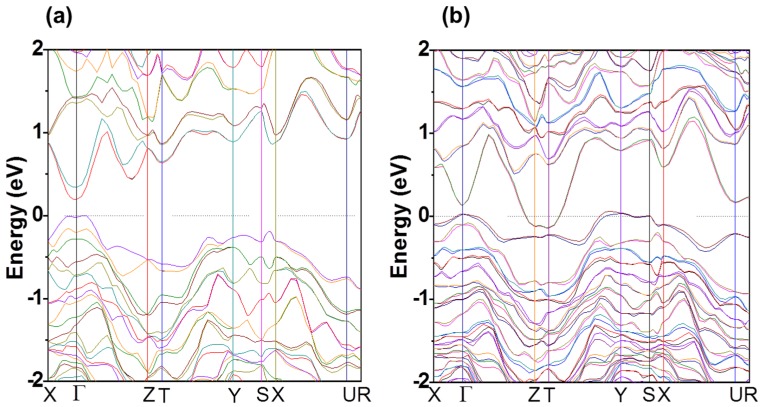
Electronic band structure of (**a**) In_4_Se_3_ and (**b**) In_4_Se_3-δ_ (δ = 0.25). Reproduced with permission from AIP Publishing LLC, 2009 [21].

A band structure calculation using density functional theory for In_4_Se_3_ and In_4_Se_3−δ_ (δ = 0.25) crystals was performed to understand the electronic ground states and the Se-deficiency effects [21]. The band structure of In_4_Se_3_ shows that the ground state of the compound is a direct band gap semiconductor (Δ= 0.2 eV), as shown in Figure 4a. The actual energy gap of In_4_Se_3_ is expected to be greater than 0.2 eV, since the band gap calculated by Generalized Gradient Approximation (GGA) is usually smaller than the experimental value [23]. The analysis of the wave function character shows that the 5*s* orbitals of the interstitial In atoms interact with the 4*p* orbitals of the neighboring Se atoms around the valence band edge, indicating that the interstitial In atoms have a notable effect on the band gap formation.

In contrast to the semiconducting In_4_Se_3_ crystal, the Se-deficient In_4_Se_3−δ_ (δ = 0.25) crystal shows a semimetallic band structure with both hole and electron carriers which is shown in Figure 4b. The semimetallic band character of the compound qualitatively describes the metallic evolution of the electrical transport properties for the Se deficient compounds. The dispersive electron bands near the Fermi level along the Γ–Z and T–Y symmetry lines indicate that the electric conduction path of In_4_Se_3−δ_ is along the *c*-axis, which induces quasi-one-dimensional electronic transport. In the case of the hole bands, however, flat band dispersions exist along the X–Γ and Y–S symmetry lines, indicating that the holes in the *b*-direction are highly localized. In addition, an extremely small hole density is anticipated for the small and flat hole band near the Γ, Y, and S points. Because of the low density of the localized holes, the electrical transport properties are mainly dominated by electron carriers, which is consistent with *n*-type Seebeck coefficients. The anisotropic energy band structure of the crystal implies significant anisotropy in the electrical transport properties, a strong van der Waals interaction along the *a*-axis, a highly localized hole band along the *b*-axis, and a dispersive electron band along the *c*-axis. The anisotropic band structure ensures that the low-dimensional transport of carriers and the localized hole band prevents the electron-hole compensation effect in Seebeck coefficients. The low-dimensional electronic transport is attributed to the large Seebeck coefficient for those compounds.

#### 2.2.2. Thermoelectric Properties of In_4_Se_3−δ_ Bulk Crystalline Materials

**Figure 5:**
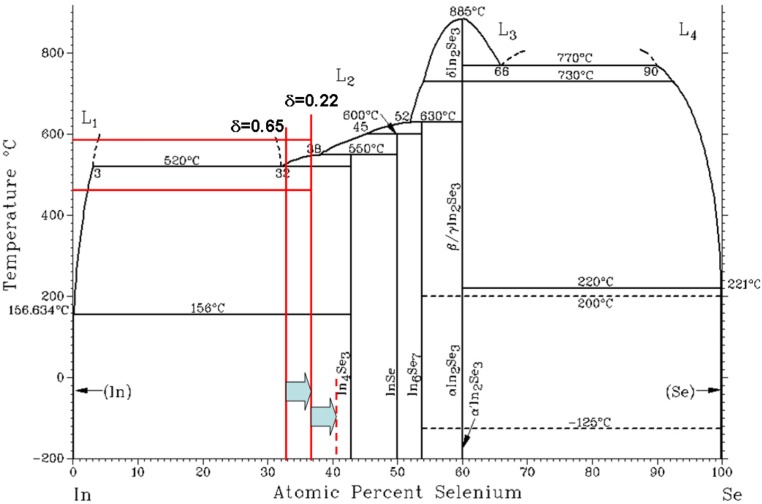
Binary phase diagram of In–Se. The starting compositions of grown crystals of In_4_Se_3−δ_ (δ = 0.65 and 0.22) are of In_67_Se_33_ and In_63_Se_37_, respectively. Excess In as much as 4 at.% segregated at the top of the crystal ingots. Red horizontal line represents the melting (590 °C) and crystallizing temperature (460 °C) range of δ = 0.22 compound. Reproduced with permission from Nature publishing Group, 2009 [15].

In short, In_4_Se_3−δ_ has the ingredients for high *ZT* in terms of the band structure; anisotropic band dispersion with hole localization along the *b*-axis and significant electron dispersion along the *c*-axis, and low thermal conductivity. In order to reveal the Peierls distortion, crystalline materials are needed for reduced dimensionality. The In_4_Se_3−δ_ crystal ingots were grown by the Bridgeman method. By comparing the initial and final In/Se concentrations, we found that the excess In about 4 at.% does not incorporate the crystallization at this concentration range during the crystal growth as shown in Figure 5. Because excess In was floated in the upper part of the crystal during the crystal growth, we eliminated the upper part (≥2 mm) of the grown crystals.

**Figure 6:**
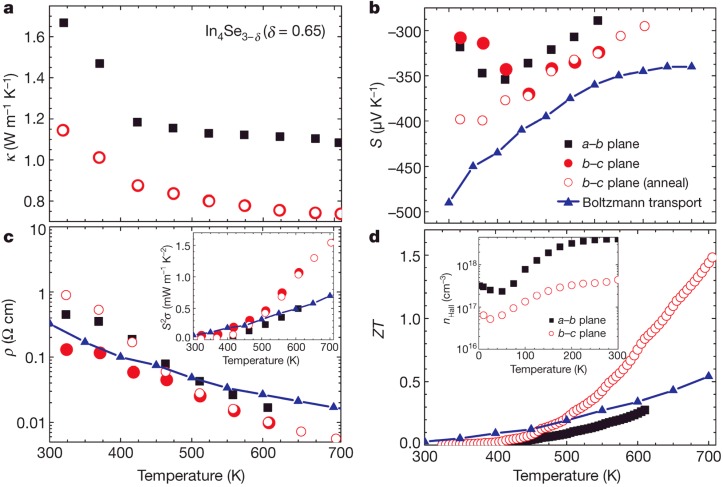
Thermoelectric properties of bulk crystalline In_4_Se_3−δ_ (δ = 0.65); (**a**) thermal conductivity, (**b**) Seebeck coefficient, (**c**) electrical resistivity (inset shows the power factor), and (**d**) Dimensionless figure-of-merit (inset shows the Hall carrier concentration). Reproduced with permission from Nature publishing Group, 2009 [15].

Figure 6 shows the thermoelectric properties of In_4_Se_2.35_ (δ = 0.65) for both orientations of the growth direction (*ab*-plane, black square) and perpendicular to the growth direction (*bc*-plane, red circle) with theoretical Boltzmann transport calculation result [15]. As shown in Figure 6a, the thermal conductivity κ(T) of In_4_Se_2.35_ is very low (≤1.2 W·m^−1^·K^−1^ at 300 K) along the *bc*-plane (covalent bonding layer), and it decreases with increasing temperatures (0.74 W·m^−1^ K^−1^ at 705 K). The thermal conductivities before and after heat treatment (450 °C for 24 h) are almost identical along the *bc*-plane (open red circle). Importantly, the thermal conductivity of covalent bonding layer (*bc*-plane) is lower than that of the van der Waals bonding layer (*ab*-plane) which is in contrast to the conventional wisdom. This indicates that there is a thermal conductivity reduction mechanism along the covalent bonding layer or charge conducting layer (*bc*-plane).

The Seebeck coefficient S(T) and electrical resistivity ρ(T) show that the In_4_Se_3−δ_ (δ ≤ 0.65) are *n*-type materials with negative Seebeck coefficient as shown in Figure 6b,c. The dimensionless figure of merit *ZT* shown in Figure 6d, reaches the remarkably high value 1.48 for In_4_Se_2.35_ (δ = 0.65) at 705 K along the *bc*-plane. In the case of In_4_Se_2.78_ (δ = 0.22), *ZT* reaches 1.1 at the same temperature. The Hall carrier concentration $n_{\text{Hall}}$ of the In_4_Se_2.35_ (δ = 0.65) crystal for both crystal orientations along the *ab*- and *bc*-plane, which is determined by Hall resistivity measurements, estimated to be 4 × 10^18^ cm^−3^ and 4 × 10^17^ cm^−3^ at 300 K, respectively, as shown in the inset of Figure 6d. The carrier concentrations for those compounds of In_4_Se_3−δ_ (7 × 10^18^ cm^−3^ along the *ab*-plane for δ = 0.22) are slightly lower than the optimum carrier concentration (10^19^~10^20^ cm^−3^) for narrow band-gap semiconductors [24].

#### 2.2.3. Peierls Distortion of Bulk Crystalline In_4_Se_3−δ_

**Figure 7:**
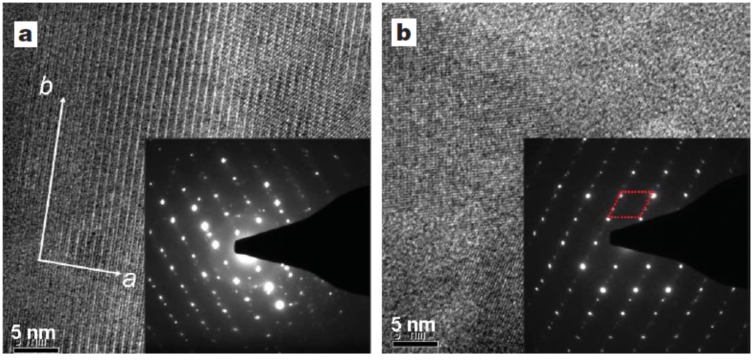
High-resolution TEM images and electron diffraction patterns of bulk crystalline In_4_Se_2.78_ (δ = 0.22) in (**a**) the *ab*-plane and (**b**) the *bc*-plane. Reproduced with permission from Nature publishing Group, 2009 [15].

The criteria of the existence of Peierls distortion is the lattice distortion and commensurate charge modulation with the lattices. The lattice distortion can be observed by the high-resolution transmission electron microscopy (HRTEM) images and electron diffraction patterns [15]. Figure 7a shows the HRTEM image of the *ab*-plane of In_4_Se_2.78_ (δ = 0.22) crystal. The Bragg spots of the electron diffraction pattern in the *ab*-plane (inset of Figure 7a) accompany small superstructure peaks in the chain direction. Quasi-one-dimensional Bragg spots and secondary superstructure peaks indicate the lattice distortion along the chain direction. Figure 7b shows the HRTEM image of the cross-sectional plane of the In_4_Se_2.78_ (δ = 0.22) crystal ingot. There are several grain boundaries between the stripe and checkerboard patterns with arbitrary orientation: the stripe and checkerboard patterns are considered to be the *ac*- and *bc*-planes respectively. The grain boundaries of different crystal orientations are about 20 nm or less. The electron diffraction pattern of the *bc*-plane shows a rhomboidal Bragg diffraction pattern with one-dimensional superlattice peaks between the bright Bragg peaks (shown in the inset to Figure 7b) indicating the presence of a Peierls instability along the *b*-axis direction.

**Figure 8:**
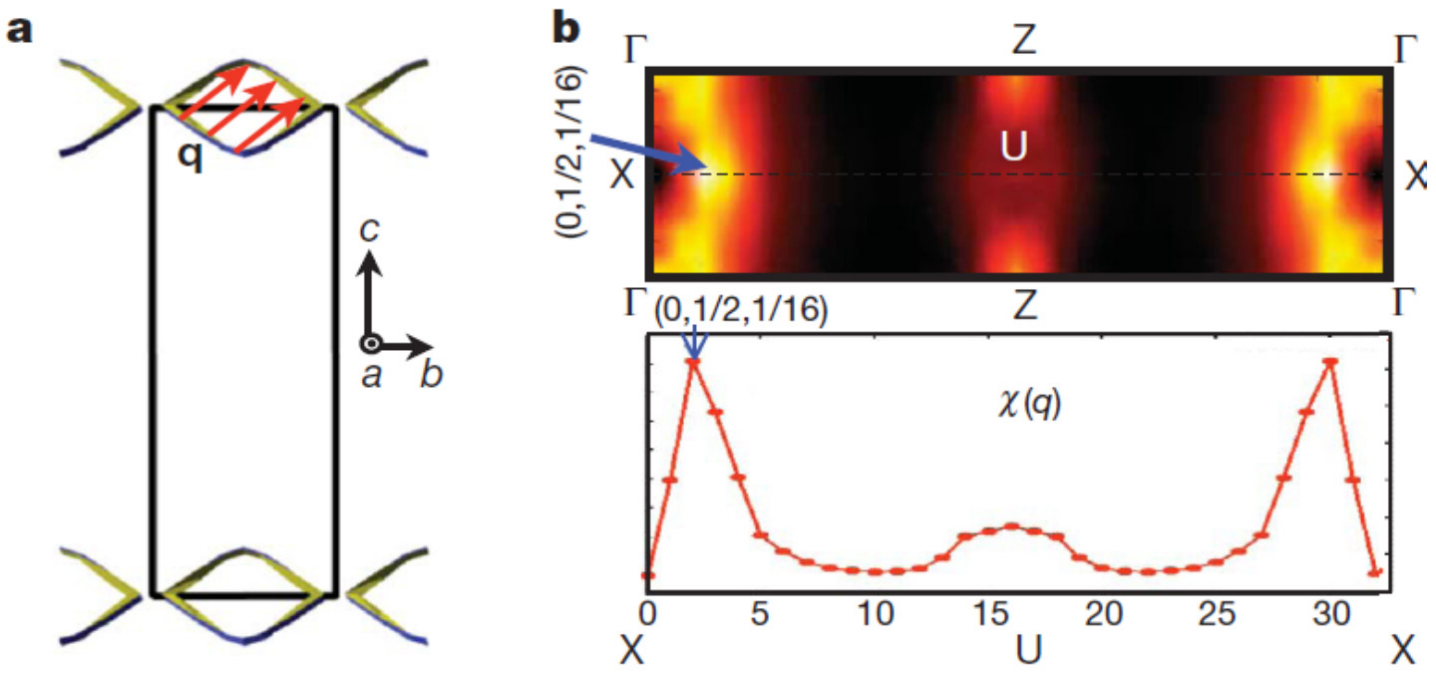
Fermi surface and generalized electron susceptibility χ(q) of In_4_Se_3−δ_ (δ = 0.25). (**a**) Fermi surface of In_4_Se_3−δ_ (δ = 0.25) in the *bc*-plane (blue and green lines). Black square is the first Brillouin zone. Fermi nesting vector q (red arrow) is defined in the closed Fermi surface. (**b**) Generalized electron susceptibility χ(q) along the X(0, 1/2, 0)–U(0, 1/2, 1/2) symmetry line (top). Reproduced with permission from Nature publishing Group, 2009 [15].

The Peierls instability of this material is also suggested by theoretical considerations, once we explicitly include the Se vacancies in the calculations. We calculated the generalized electron susceptibility χ(q) from the band structure of In_4_Se_3−δ_ (δ = 0.25) which is shown along the X–U symmetry line in Figure 8b [15]. The sharp peak at the (0, 1/2, 1/16) point can be understood from the quasi-one-dimensional Fermi surface (FS) nesting in the *bc*-plane shown in Figure 8a. It consists of two smooth diamond-shaped FS’s located at the upper and lower zone boundaries of the first Brillouin zone (BZ). There is a well-defined commensurate nesting vector (red arrow) defined in the *bc*-plane which can result in a Charge Density Wave (CDW) instability once the electron-phonon or electron–electron correlations are incorporated in a calculation going beyond Local Density Approximation (LDA). Because of the long range lattice modulation along the *c*-direction, the density wave instability is closely connected to the quasi-one-dimensional Peierls instability of the chain-like structure along the *b*-axis of this material. The electron diffraction patterns shown in the inset of Figure 7b are consistent with this FS nesting behavior. The small faint peaks between the Bragg spots indicate the doubling superstructure in the *b*-direction, while the long-range modulation in the *c*-direction cannot be seen, probably due to the experimental resolution.

Thermal transport properties shown in Figure 6a also suggest the presence of a charge density wave. Notice that the thermal conductivity in the *bc*-plane is lower than that in the *ab*-plane in the In_4_Se_2.35_ crystal, which is at first sight surprising since the bonding in the *bc*-plane is weaker than in the *ab*-plane. The in-plane (*bc*-plane) lattice distortion driven by the CDW lowers the thermal conductivity. In addition, the Hall carrier concentration is anisotropic with respect to crystal orientations. In usual cases, the carrier density is isotropic with respect to crystal orientations. However, in this case, the carrier density along the *bc*-plane is lower than those of the *ab*-plane, which is caused by the CDW along the *bc*-plane. Because of the CDW gap opening, the itinerant carrier density is decreased along the *bc*-plane. Those facts and the S(T) and ρ(T) shown in Figure 6b,c, suggest the formation of a charge density at a temperature higher than 705 K.

Thermoelectric investigations on In_4_Se_3−δ_ crystals suggest that bulk low-dimensional layered materials with strong electron-phonon coupling, such as Peierls or charge density wave instabilities, are promising candidates for new thermoelectric materials. Compared to other realizations of high *ZT* *n*-type thermoelectric materials through nano-scale phase separation [25,26], the CDW mechanism for high *ZT* has the advantage of being realized as an intrinsically bulk phenomena without artificial control of nanoscale phase segregation. This resulted in an exceptionally high *ZT* (1.48 at 705 K) in compounds with high chemical stability and good mechanical properties that can be combined with well-established *p*-type thermoelectrics to produce highly efficient thermoelectric power generation modules.

#### 2.2.4. Boltzmann Transport Result of In_4_Se_3−δ_

To understand the microscopic origin of the exceptional properties of this material we carried out first principles transport calculation of In_4_Se_3_ using BoltzTraP program. Calculations of the angle averaged transport coefficients, within the rigid band approximation can account for the experimental trends and confirm the potential of In_4_Se_3−δ_ as a thermoelectric material. Using a chemical potential μ = 0.22 eV and a scattering time τ = 2.2 × 10^−14^ s, we obtained an electron concentration 1 × 10^18^ cm^−3^ and high Seebeck coefficient (−360 μV/K at 600 K) and which is comparable to the observed experimental value. The calculated temperature-dependent behavior of S(T) and ρ(T) can qualitatively reproduce the experimental data as shown in Figure 6b,c (blue line and triangle). Using a constant value of the angle averaged lattice thermal conductivity $\kappa_{ph}$ = 0.8 W·m^−1^·K^−1^, we can account for the temperature dependence of the power factor and *ZT* as shown in the inset of Figure 6c,d, respectively. Within the rigid band approximation, the carrier density of those compounds is not yet optimal.

**Figure 9:**
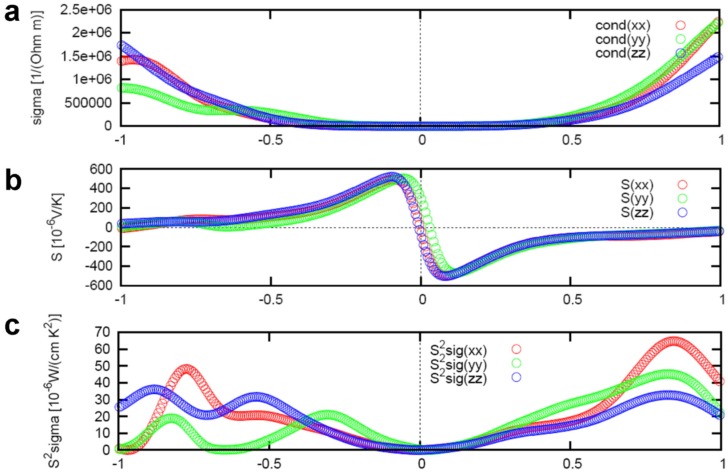
Boltzmann transport calculation result of the (**a**) electrical conductivity σ, (**b**) Seebeck coefficient S, and (**c**) power factor $S^{2}\text{σ}$ with respect to chemical potential μ of the In_4_Se_3−δ_ (δ = 0.25) compound under fixed conditions of temperature *T* = 600 K and relaxation time of scattering τ = 2.2 × 10^−14^ s. Reproduced with permission from Nature publishing Group, 2009 [15].

Figure 9 represents the anisotropic Boltzmann transport calculation results of electrical conductivity σ, Seebeck coefficient S, and power factor $S^{2}\text{σ}$ with respect to chemical potential μ of the In_4_Se_3_ compound. The positive (negative) chemical potential indicates the electron (hole) doping, respectively. The power factor is maximum near μ = 0.8 eV due to significant increase of the electrical conductivity at the high electron doping range. The Boltzman transport result as shown in Figure 6 used the chemical potential of μ = 0.22 eV which is far from the optimum value for high power factor. Therefore, the power factor of the presented compound In_4_Se_3-δ_ (δ = 0.64, *ZT* = 1.48 at *T* = 705 K) can be further increased by increasing electron doping (up to μ = 0.8 eV) level.

### 2.3. Enhancement of ZT by Increasing Chemical Potential

#### 2.3.1. Cation Doping in Polycrystalline In_3.9_*M*_0.1_Se_2.95_

In the crystal structure of In_4_Se_3_, the average charge of indium within the layer (In1, In2, and In3 site) is ~1.67+ while that of indium between the layers (In4 site) is 1+. In the solid state chemistry point of view, the carrier concentration in In_4_Se_3_ based materials could be controlled through replacing the indium by other metals because of their different valence states. It is likely that the In4 site replacement is more susceptible than the other indium site replacements because of strong covalent-ionic interaction between indium at In1, In2, and In3 sites and selenium within the layer. A formation energy calculation using the density functional theory (DFT) was carried out in order to predict the most preferential indium site among In1, In2, In3, and In4 sites for each substituted metal atom we studied here. The formation energies of In_4−*x*_M*_x_*Se_3_ (*x* = 0.25) were calculated at each possible indium site as follows:

In_16_Se_12_ + M → In_15_MSe_12_ (*x* = 0.25) + In (excess) (2)

*E*_f_ = *E*_t_ (In_15_MSe_12_) – *E*_t_ (In_16_Se_12_) + *E*_t_ (In) – *E*_t_ (M) (3)

where *E*_f_ is the formation energy and *E*_t_ the total energy of each material.

**Figure 10:**
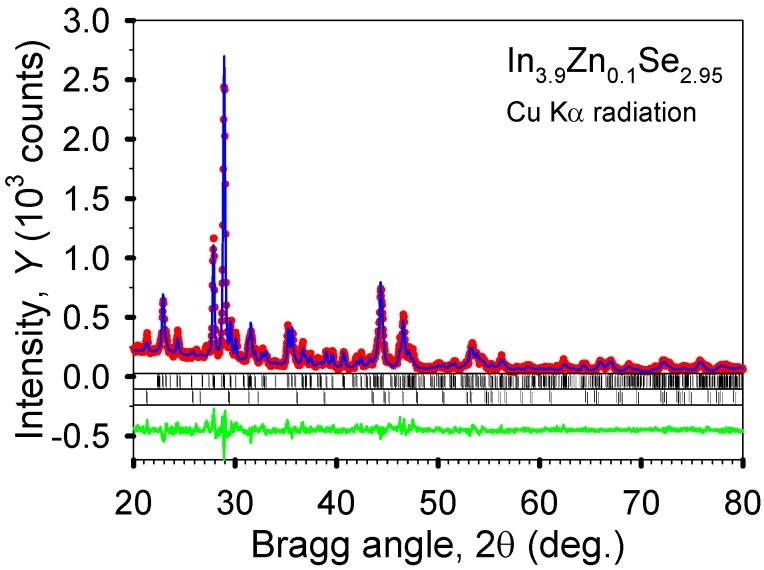
The observed (dots) and calculated (line) powder diffraction patterns of polycrystalline In_3.9_Zn_0.1_Se_2.95_ after the completion of Rietveld refinements. The upper sets of vertical bars located just below the plots of the observed and calculated intensities indicate the calculated positions of the Bragg peaks of the majority 4:3 In–Se phase, while the lower sets of bars correspond to the calculated positions of the Bragg peaks of the 1:1 In–Se impurity. The difference, *Y*_obs_ – *Y*_calc_, is shown at the bottom of the plot.

The formation energies of In_4−*x*_*M_x_*Se_3_ (*x* = 0.25) at each indium site are tabulated in Table 2. This shows that the metal substitution at the In4 site is energetically most favorable for M = Na, Ca, and Pb while other sites are most preferable for M = Sn (In2 site) and M = Zn and Ga (In3 site). In Figure 10, the crystal structure of In_3.9_Zn_0.1_Se_2.95_ was refined by a full profile Rietveld refinement technique using an LHPM-Rietica program. It can be estimated from the refinement result that the major phase (>97 wt%) is the 4:3 In–Se phase and the minor phase is the 1:1 In–Se phase (<3 wt%). Regarding the metallic radii of the elements (Na, Ca, Zn, Ga, Sn, Pb) studied here, compared to the metallic radius of 1.58 Å for indium, the largest and smallest radius are 1.97 Å for Ca and 1.36 Å for Zn, respectively. A certain amount of metal substitution for indium in the 4:3 indium selenide should affect the variation of the lattice parameters. However, the lattice parameters of In_4−*x*_M*_x_*Se_2.95_ samples are fairly similar to each other. Also, these are almost same as that of In_4_Se_2.95_ (*a* = 15.281(1) Å, *b* = 12.301(1) Å, *c* = 4.075(1) Å). This indicates that the small amount of metal doping below 2.5 at.% for indium on In_4_Se_2.95_ shows a negligible influence on the lattice parameters.

**Table 2:** Formation energies in eV at each indium site for In_4−*x*_*M_x_*Se_3_ (*x* = 0.25).

In-sites	Na	Ca	Zn	Ga	Sn	Pb
In1	−0.430	−1.913	0.065	−0.423	0.157	0.173
In2	−0.776	−1.831	0.215	−0.422	0.022	0.065
In3	−0.527	−1.913	0.011	−0.518	0.139	0.234
In4	−1.716	−2.618	0.246	−0.243	0.056	−0.144

**Figure 11:**
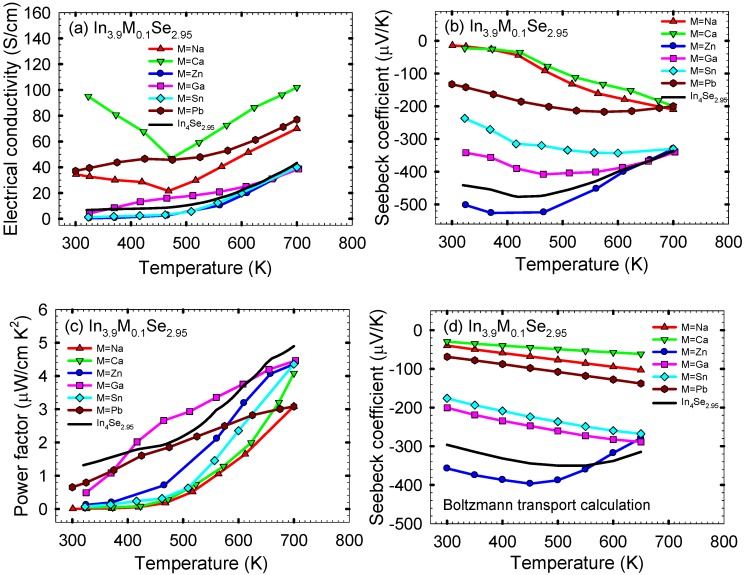
Temperature dependence of (**a**) electrical conductivity, (**b**) Seebeck coefficient, (**c**) power factor, and (**d**) calculated Seebeck coefficient by Boltzmann transport equation of polycrystalline samples of In_3.9_M_0.1_Se_2.95_ (M = Na, Ca, Zn, Ga, Sn, Pb) and In_4_Se_2.95_. Reproduced with permission from AIP Publishing LLC, 2011 [27].

The thermoelectric properties of polycrystalline In_3.9_M_0.1_Se_2.95_ (M = Na, Ca, Zn, Ga, Sn, Pb) samples were investigated in order to explore the effect of cationic (metallic) substitution on thermoelectric performance of In_4_Se_2.95_ [21] which was reported to the optimal Se-deficient composition for a high *ZT* among In_4_Se_3-*x*_ polycrystalline materials. Figure 11a shows the electrical conductivity σ as a function of temperature for polycrystalline In_3.9_*M*_0.1_Se_2.95_ (M = Na, Ca, Zn, Ga, Sn, Pb) and In_4_Se_2.95_ samples. Compared to the electrical conductivity at ~320 K of ~7 S/cm for In_4_Se_2.95_, those at ~320 K for In_3.9_Na_0.1_Se_2.95_, In_3.9_Ca_0.1_Se_2.95_, In_3.9_Zn_0.1_Se_2.95_, In_3.9_Ga_0.1_Se_2.95_, In_3.9_Sn_0.1_Se_2.95_, and In_3.9_Pb_0.1_Se_2.95_ are ~33, ~95, ~0.5, ~4, ~1, and ~37 S/cm, respectively. The Hall mobility μ_*H*_ can be expressed as ${\text{μ}}_{H}=\text{σ}/(n_{H}e)$, where σ is the electrical conductivity, *n_H_* the Hall carrier concentration, and *e* the electron charge.

The Hall carrier concentrations of In_3.9_M_0.1_Se_2.95_ polycrystalline samples are measured at room temperature (Table 3). Thus, compared to the room temperature Hall mobility of ~242.7 cm^2^/V·s for In_4_Se_2.95_, those for In_3.9_Na_0.1_Se_2.95_, In_3.9_Ca_0.1_Se_2.95_, In_3.9_Zn_0.1_Se_2.95_, In_3.9_Ga_0.1_Se_2.95_, In_3.9_Sn_0.1_Se_2.95_, and In_3.9_Pb_0.1_Se_2.95_ are ~17.2, ~6.8, ~36.8, ~45.5, ~8.3, and ~45.3 cm^2^/V·s, respectively (Table 3). For most of our samples the electrical conductivities increase with increasing temperature indicative of semiconducting behavior. However, the sample of In_3.9_Na_0.1_Se_2.95_ and In_3.9_Ca_0.1_Se_2.95_ show metal-semiconductor-like transitions at ~470 K, which probably comes from a thermal stability issue (*i.e.*, decomposition of the compounds at a certain temperature). Compared to ~43 S/cm at ~700 K for In_4_Se_2.95_, the respective electrical conductivities at ~700 K for M = Na, Ca, Zn, Ga, Sn, and Pb are ~70, ~102, ~40, ~39, ~40, and ~77 S/cm. Especially, it is noted that the electrical conductivity at ~320 K for In_3.9_Ca_0.1_Se_2.95_ is ~15 times higher than In_4_Se_2.95_ indicating that the Ca substitution should be effective for increasing the carrier concentration. This is supported by the room temperature electron concentration of 8.7 × 10^19^ cm^−3^ for In_3.9_Ca_0.1_Se_2.95_ compared to that of 1.74 × 10^17^ cm^−3^ for In_4_Se_2.95_ (Table 3). The most preferential site for metal substitution in the In_3.9_M_0.1_Se_2.95_ compounds (Table 2) may be associated with the carrier concentration. The Na, Ca, and Pb substituted compounds show much higher room temperature electron concentrations than the Zn, Ga, and Sn substituted ones. This implies that metal substitutions at the In4 site are more effective to increase the carrier concentration than those at other indium sites.

**Table 3:** Room temperature carrier concentrations and Hall mobilities of the polycrystalline samples of In_3.9_M_0.1_Se_2.95_ (M = Na, Ca, Zn, Ga, Sn, Pb) and In_4_Se_2.95_.

Composition	Electron Concentration (cm^−3^)	Hall Mobility (cm^2^/V s)
In_4_Se_2.95_	1.7 × 10^17^	242.7
In_3.9_Na_0.1_Se_2.95_	1.2 × 10^19^	17.2
In_3.9_Ca_0.1_Se_2.95_	8.7 × 10^19^	6.8
In_3.9_Zn_0.1_Se_2.95_	8.5 × 10^16^	36.8
In_3.9_Ga_0.1_Se_2.95_	5.5 × 10^17^	45.5
In_3.9_Sn_0.1_Se_2.95_	7.5 × 10^17^	8.3
In_3.9_Pb_0.1_Se_2.95_	5.1 × 10^18^	45.3

Figure 11b shows the temperature dependent Seebeck coefficient *S* of the polycrystalline samples of In_3.9_M_0.1_Se_2.95_ (M = Na, Ca, Zn, Ga, Sn, Pb) and In_4_Se_2.95_. All samples exhibit negative values of Seebeck coefficient indicating the electron as a major charge carrier. In particular, the samples of In_3.9_Na_0.1_Se_2.95_ and In_3.9_Ca_0.1_Se_2.95_ exhibit significantly lower absolute values of Seebeck coefficient over the measured temperature range compared to In_4_Se_2.95_. For instance, the absolute value of Seebeck coefficient at ~320 K for In_3.9_Ca_0.1_Se_2.95_ is ~1/22 times smaller than In_4_Se_2.95_ when its electrical conductivity at the same temperature is ~15 times higher than In_4_Se_2.95_. We can expect that the calcium substitution on In_4_Se_2.95_ should lower its power factor. For degenerate semiconductors the Seebeck coefficient is inversely proportional to the carrier concentration according to the below equation [22]:

(4) $$S=\left(\frac{8\pi^{2}k_{B}^{2}T}{3eh^{2}}\right)\cdot m_{d}^{*}\cdot {\left(\frac{\pi }{3n}\right)}^{2/3}$$

where *k_B_* is the Boltzmann constant, *e* the electron charge, *h* the Planck constant, m_d_* the effective mass, and *n* the carrier concentration.

We utilized a Boltzmann transport equation (BTE) using a rigid band approximation in order to calculate the Seebeck coefficient as a function of temperature at the experimentally measured each carrier concentration of the polycrystalline samples of In_3.9_M_0.1_Se_2.95_. In this calculation, the band gap of 0.4 eV was taken into account because of the empirical band gap of ~0.4 eV for polycrystalline In_4_Se_2.95_ according to the equation *E_g_* = 2*e*│*S*_max_│*T*_max_ where *E_g_* is the band gap, *e* the electron charge, │*S*_max_│ the maximum absolute value of Seebeck coefficient, and *T*_max_ the temperature at which the maximum occurs (*i.e.*, │*S*_max_│~ 480 μV/K and *T*_max_ ~ 420 K for polycrystalline In_4_Se_2.95_). The calculated temperature dependent Seebeck coefficients of In_3.9_M_0.1_Se_2.95_ compounds using BTE are shown in Figure 11d. It is noted that for each sample the temperature dependence of Seebeck coefficient by BTE are nearly similar to those by experimental measurement. Thus, this strongly indicates that the cationic (metallic) substitution on In_4_Se_2.95_ decreases (or increases) its Seebeck coefficient in absolute magnitude with increasing (or decreasing) the electron concentration because of the substituted metals located at indium sites in light of the formation energy calculation results and their role on the control of carrier concentration. The quantum confinement effect of quasi-one-dimensional chains on the Seebeck coefficient for the 4:3 indium selenide may not be either somewhat weakened or lost by the metal substitutions for indium sites in the lattice of the 4:3 indium selenide.

The corresponding temperature dependent power factors σ*S*^2^ of the polycrystalline samples of In_3.9_M_0.1_Se_2.95_ (M = Na, Ca, Zn, Ga, Sn, Pb) and In_4_Se_2.95_ are plotted in Figure 11c. For all samples the power factor increases with increasing temperature, which is similar to In_4_Se_2.95_. As expected, all the samples show lower power factors than ~1.3 μW/cm·K^2^ at ~320 K for polycrystalline In_4_Se_2.95_. It is noted that the cationic (metallic) replacement on In_4_Se_2.95_ lowers its power factor due to the larger reduction on the absolute value of Seebeck coefficient with the smaller increase in the electrical conductivity.

**Figure 12:**
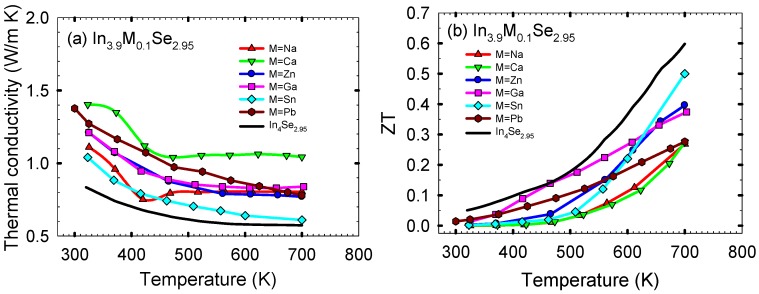
(**a**) Temperature dependence of thermal conductivity, and (**b**) the thermoelectric figure of merit of polycrystalline samples of In_3.9_M_0.1_Se_2.95_ (M = Na, Ca, Zn, Ga, Sn, Pb) and In_4_Se_2.95_. Reproduced with permission from AIP Publishing LLC, 2011 [27].

Figure 12a shows the thermal conductivity κ as a function of temperature for the polycrystalline samples of In_3.9_M_0.1_Se_2.95_ (M = Na, Ca, Zn, Ga, Sn, Pb) and In_4_Se_2.95_. The thermal conductivities of In_3.9_M_0.1_Se_2.95_ are higher than that of In_4_Se_2.95_ over the measured temperature range. Normally, the thermal conductivity is the sum of the electronic thermal conductivity κ_elec_ and the lattice thermal conductivity κ_latt_. The κ_elec_ can be calculated from the Wiedemann-Franz law, κ_elec_ = *L*σ*T*, where *L* is the Lorenz number. Subtracting the electronic term from the total thermal conductivity one obtains an estimate of the lattice thermal conductivity of a sample. However, for all the samples in this work their lattice thermal conductivities are approximately same as their total thermal conductivities because their electronic thermal conductivities are quite low (*i.e.*, for In_3.9_Na_0.1_Se_2.95_ the electronic thermal conductivity κ_elec_ at ~320 K is ~0.03 W/m·K when we take the Lorenz number *L* as 2.45 × 10^−8^ V^2^/K^2^ for metals). It is noted that the room temperature lattice thermal conductivity of In_4_Se_2.95_ is comparable to that of nanostructured bulk thermoelectric materials such as Ag_1−*x*_Pb_18_SbTe_20_ and Bi_*x*_Sb_2−*x*_Te_3_ (*i.e.*, ~0.8 W/m·K for Ag_1−*x*_Pb_18_SbTe_20_ and ~0.6 W/m·K for Bi_*x*_Sb_2−*x*_Te_3_) [25,28].

In general, the alloying/solid solution (point defect scattering) in a compound should decrease its lattice thermal conductivity further compared to the compound having no point defect. However, in the case of the 4:3 indium selenide, the point defects do not reduce the lattice thermal conductivities further compared to that of polycrystalline In_4_Se_2.95_, and those of In_3.9_M_0.1_Se_2.95_ are rather higher than that of In_4_Se_2.95_. The reasons should be as follows: the 4:3 indium selenide is a charge density wave system in which a strong electron-phonon coupling breaks the translational symmetry of lattice (*i.e.*, Peierls distortion) [7,15]. We speculate that the cationic (metallic) substitution on the bulk low-dimensional layered In_4_Se_3−*x*_ crystal either significantly weakens or removes the effect of the Peierls distortion on the reduction in lattice thermal conductivity probably due to the metal substitution at the indium sites. Thus, the main reduction in the lattice thermal conductivity of polycrystalline In_4−*x*_*M*_*x*_Se_2.95_ does not result from the Peierls distortion while the lattice thermal conductivity of In_4_Se_2.95_ is quite suppressed mainly by the Peierls distortion. This can explain why the point defects do not further reduce the lattice thermal conductivity of In_4_Se_2.95_.

Finally, the dimensionless thermoelectric figure of merit *ZTs* for the polycrystalline samples of In_3.9_M_0.1_Se_2.95_ (M = Na, Ca, Zn, Ga, Sn, Pb) and In_4_Se_2.95_ are plotted in Figure 11b. For all samples, the *ZT* increases with increasing temperature. Compared to the *ZT* of ~0.05 at ~320 K for In_4_Se_2.95_, those at ~320 K for In_3.9_Na_0.1_Se_2.95_, In_3.9_Ca_0.1_Se_2.95_, In_3.9_Zn_0.1_Se_2.95_, In_3.9_Ga_0.1_Se_2.95_, In_3.9_Sn_0.1_Se_2.95_, and In_3.9_Pb_0.1_Se_2.95_ are ~0.0003, ~0.0012, ~0.0033, ~0.013, ~0.0022, and ~0.020, respectively. Compared to the *ZT* of ~0.60 at ~700 K for In_4_Se_2.95_, the respective *ZTs* at ~700 K are ~0.27, ~0.27, ~0.40, ~0.37, ~0.50, and ~0.28.

Thus, the indium replacement by various metals such as Na, Ca, Zn, Ga, Sn, and Pb on In_4_Se_2.95_ degrades the thermoelectric performance of In_4_Se_2.95_ mainly due to the reduction in the power factor and the increase in the lattice thermal conductivity by either quite weak Peierls distortion or no Peierls distortion by the metal substitutions at the indium sites. For the 4:3 indium selenide the quasi-one-dimensional In–Se chain in the CDW plane is a main factor for the thermoelectric performance because of the enhancement on the Seebeck coefficient due to the In–Se chain (quantum confinement) as well as the reduction on the lattice thermal conductivity due to the Peierls distortion in the CDW plane. In our work, the metal substitutions change the normal physical trend for thermal properties of In_4_Se_2.95_ while they maintain the same trend for electrical properties of In_4_Se_2.95_ with different carrier concentrations. Unlike the cationic substitution, the anionic substitution on the 4:3 indium selenide could be promising for high efficient thermoelectric properties if the above mentioned roles of the chain in the CDW plane kept unchanged or even improved with the anionic substitution. The substitution of isovalent and nonisoelectronic elements for selenium on the 4:3 indium selenide will be investigated in the future.

#### 2.3.2. High *ZT* over a Wide Temperature Range in Halogenated In_4_Se_3−*x*_*H*_0.03_ (*H* = Cl, Br, and I) Bulk Crystals

In spite of high *ZT* (1.48) in In_4_Se_3−δ_ crystal, two challenges remain for practical applications. Firstly, the reported *ZT* could be increased further if we could increase the carrier concentration of the In_4_Se_3−δ_ crystals because it is far from the carrier concentration of a heavily doped semiconductor (on the order of 10^19^ cm^−3^) that is generally considered to be optimal for thermoelectric materials. Secondly, *ZT* decreases significantly as the temperature decreases, which limits the operational temperature range to within 350~430 °C. By incorporating chlorine, a significantly increase of *ZT* (maximum *ZT* (*ZT*_max_) = 1.53) was observed over a wide temperature range in chlorine-doped In_4_Se_3−δ_Cl_0.03_ crystal mainly as a result of the increase in the electrical conductivity because of the increase of both the carrier density and Hall mobility [29].

**Figure 13:**
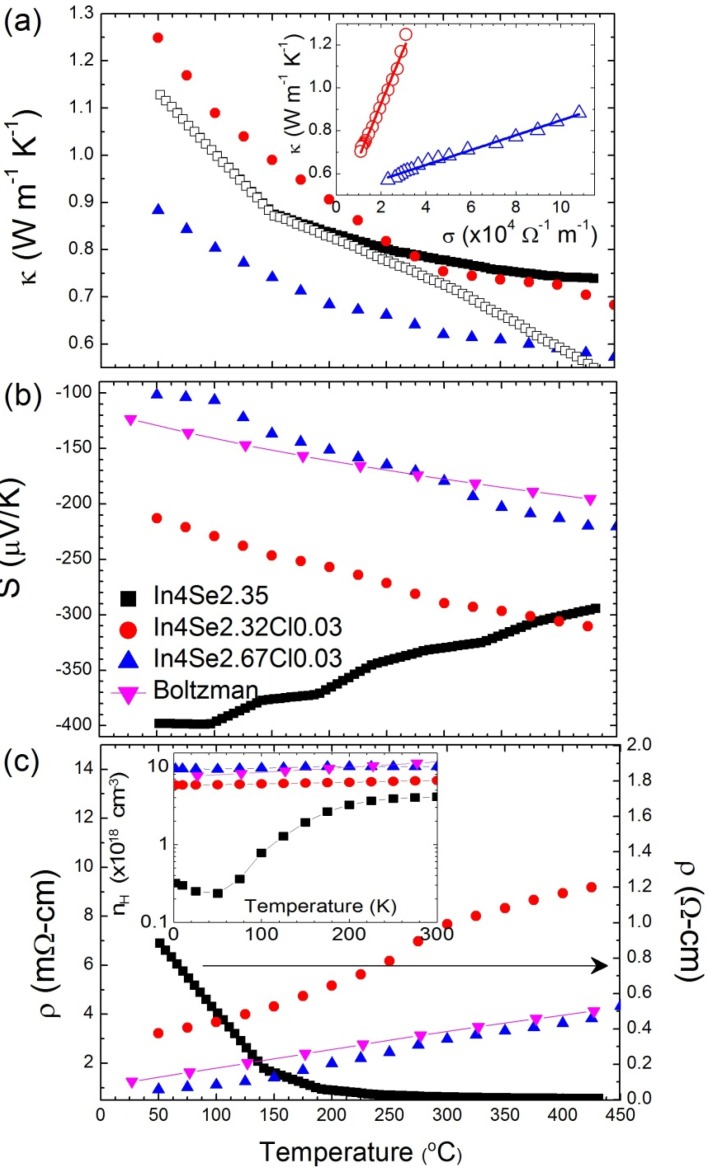
Temperature-dependent thermoelectric properties of (**a**) thermal conductivity, (**b**) Seebeck coefficient, (**c**) electrical resistivity, and Hall carrier concentration (inset of (**c**)) for In_4_Se_2.35_ [15], In_4_Se_2.32_Cl_0.03_, and In_4_Se_2.67_Cl_0.03_ bulk crystals [29]. Reprodueced with permission from WILEY-VCH Verlag GmbH & Co., 2011 [29].

Figure 13 shows the thermoelectric properties of the chlorine-doped compounds of In_4_Se_2.32_Cl_0.03_, In_4_Se_2.67_Cl_0.03_ bulk single crystals, and the previously reported [15] In_4_Se_2.35_ crystal for comparison. Because high thermoelectric performance was revealed along the *bc*-plane of the crystal containing charge density wave lattice instability, the thermoelectric properties were measured along that plane. The inherent nature of van der Waals interactions along the *a*-axis and Peierls distortion in the *bc*-plane render the low thermal conductivity. The lattice thermal conductivity of In_4_Se_2.35_ crystal is obtained by subtracting the electronic thermal conductivity from the Wiedemann-Frantz (WF) law, as shown in the open rectangular symbol in Figure 13a. However, the WF law is violated in a special case of bipolar transport [8,30]. An estimation of the lattice thermal conductivity can be made in a plot of κ *versus* σ, which shows a linear relationship, as shown in the inset of Figure 13a, because the thermal and electrical conductivities follow 1/*T* temperature dependence in both cases. The extrapolation of the thermal conductivity to zero electrical conductivity gives the lattice thermal conductivity at a high temperature limit. A rough estimation of the high temperature lattice thermal conductivity of these compounds is presented in Table 4. The estimated lattice thermal conductivities are not significantly changed by chlorine doping.

The Seebeck coefficients decrease with chlorine doping in bulk crystal, as shown in Figure 13b, because of the metallic behavior of electrical resistivity for chlorine-doped crystalline compounds, as shown in Figure 13c. When chlorine is doped into In_4_Se_3–δ_ crystal, the electrical resistivity decreases significantly (e.g., 0.92 mΩ·cm at 50 °C for In_4_Se_2.67_Cl_0.03_) to two to three orders of magnitude lower than those of the undoped crystalline compound of In_4_Se_2.35_ (0.88 Ω·cm at 50 °C). The significant decrease of ρ(T) is mainly a result of the increase in the Hall mobility as well as the increase in the carrier concentration, as shown in Table 4. The improvement of the Hall mobility by chlorine doping can be attributed to the good alignment of the crystal along the *bc*-plane parallel to the direction of the transport property measurements, as these compounds have highly anisotropic transport properties.

**Table 4:** Estimated high-temperature lattice thermal conductivity $\kappa_{ph}$ and room-temperature electrical resistivity ρ, carrier density $n_{H}$, Hall coefficient $R_{H}$, and Hall mobility $\left|\mu_{H}\right|$ of the indicated crystalline samples.

Samples	$\kappa_{ph}$ (W·m^−1^·K^−1^)	ρ (μΩ·m)	$n_{H}$ (×10^18^ cm^−3^)	$R_{H}$ (cm^3^/C)	$\left|\mu_{H}\right|$ (cm^2^·V^−1^·s^−1^)
In_4_Se_2.35_	0.54	8840	4.10	−1.52	0.017
In_4_Se_2.32_Cl_0.03_	0.43	32.24	6.70	−0.93	2.889
In_4_Se_2.67_Cl_0.03_	0.50	9.24	11.00	−0.57	6.137

The temperature-dependent behavior of the Seebeck coefficient and electrical resistivity of In_4_Se_2.67_Cl_0.03_ crystal can be reproduced by the Boltzmann transport calculation with a fixed chemical potential of μ = 0.36 eV, as shown in Figure 13b,c (line and inverse triangle symbol). It was confirmed that the chemical potential of μ = 0.22 eV is comparable to the thermoelectric properties in In_4_Se_2.35_ crystal. The power factor with respect to the chemical potential from the Boltzmann transport calculation indicated that an increase of the chemical potential to μ = 0.8 eV can increase the power factor as a result of optimization of the carrier concentration. The increase in the chemical potential by chlorine doping (0.36 eV) has an effect on the electron doping, giving rise to an increase in the electronic carrier concentration (~10^19^ cm^−3^), as shown in the inset of Figure 13c and in Table 4.

**Figure 14:**
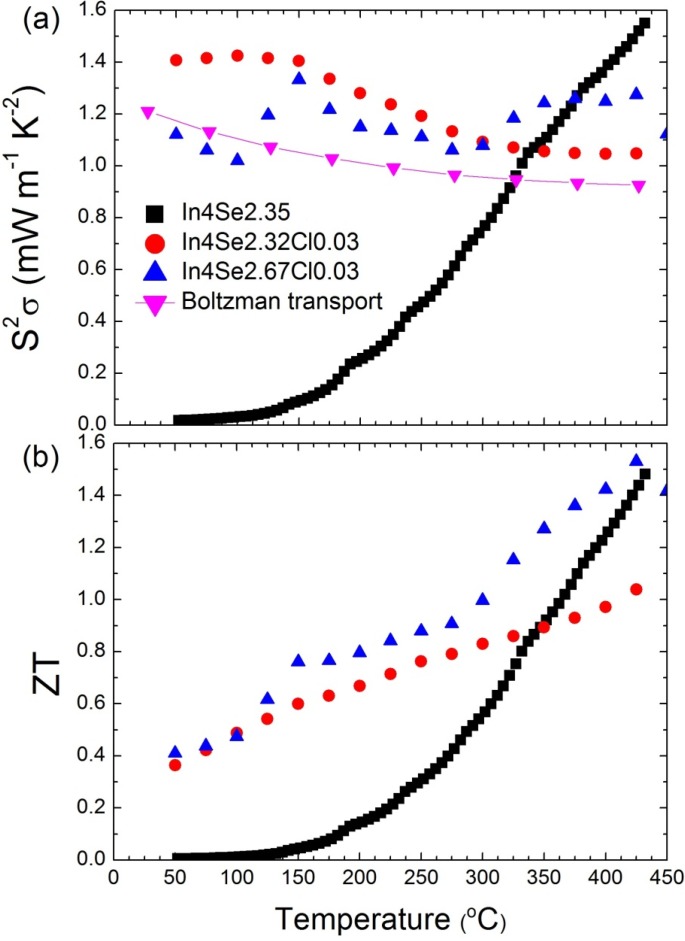
Temperature-dependent (**a**) power factor and (**b**) *ZT* value for In_4_Se_2.35_ [15], In_4_Se_2.32_Cl_0.03_, and In_4_Se_2.67_Cl_0.03_ bulk crystals [29]. Reproduced with permission from WILEY-VCH Verlag GmbH & Co., 2011 [29].

More chlorine doping does not play a significant role in the electron doping because the solubility limit of the In_4_Se_3−δ_ crystalline system is lower than 3 at.%, as confirmed by noting that the excess chlorine segregates to the surfaces of the crystal in depth profile measurements by secondary ion mass spectroscopy [31]. The metallic behavior and relatively high Seebeck coefficients in the chlorine-doped In_4_Se_3−*x*_Cl_0.03_ bulk single crystals lead to temperature-insensitive behavior of the power factor, as shown in Figure 14a. Compared to the significant decrease of $S^{2}\text{σ}$ as the temperature decreased in a previously reported case (black-closed square), the high power factors for a wide temperature range from 50 to 450 °C of chlorine-doped crystalline compounds are very important ingredients for practical applications owing to the possibility of stable power generation for such a wide temperature range. Because of a low thermal conductivity and high power factor, the *ZT* of chlorine-doped crystals exhibits a high value in a broad temperature range, as presented in Figure 14b.

The *ZT* maximum value reaches 1.53 at 425 °C, which increases *ZT* further to the previously reported In_4_Se_2.35_ crystal (*ZT* ~1.48) in *n*-type materials [15]. In addition, the room-temperature *ZT* value increases significantly from 0.005 for In_4_Se_2.35_ to 0.4 for In_4_Se_2.67_Cl_0.03_ crystals. When it is taken into account that the required *ZT* for practical power generation is approximately 0.8, the operational temperature range ($\text{Δ}T_{op}$) can be widened remarkably from 350~430 °C ($\text{Δ}T_{op}$ = 80 °C) for In_4_Se_2.35_ to 150~450 °C ($\text{Δ}T_{op}$ = 300 °C) for the In_4_Se_2.67_Cl_0.03_ crystal.

Because chlorine doping is effective to enhance thermoelectric properties over a wide temperature range, we investigated the thermoelectric properties of other halogens including fluorine, bromine, and iodine substituted In_4_Se_3−*x*_*H*_0.03_ crystals in an effort to find a high *ZT* material as well as to clarify the origin of a high *ZT* in a chlorine substituted In_4_Se_3−*x*_Cl_0.03_ crystal [32]. We show that both bromine and iodine-substituted In_4_Se_3−*x*_*H*_0.03_ crystals exhibit quite similar thermoelectric behaviors as the chlorine-substituted crystal. The fluorine-substituted crystal shows a quite different thermoelectric behavior compared to other halogens such as Cl-, Br-, and I-substituted crystals, but it is similar to the unsubstituted In_4_Se_3−*x*_ crystal.

**Figure 15:**
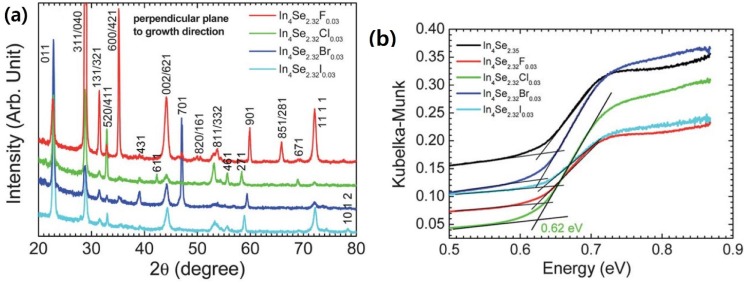
(**a**) The X-Ray diffraction patterns perpendicular planes to the crystal growth direction and (**b**) Infrared absorption spectra and of the crystals of In_4_Se_3−*x*_*H*_0.03_ (*x* = 0.68; *H* = F, Cl, Br, I) and In_4_Se_2.35_ [32].

Figure 15a shows X-ray diffraction (XRD) patterns of the crystals of In_4_Se_3−*x*_*H*_0.03_ (*x* = 0.68; H = F, Cl, Br, I). The X-ray diffraction patterns of the crystals were collected along their perpendicular planes to the crystal growth direction. The In_4_Se_3−*x*_*H*_0.03_ crystals exhibit nearly the same diffraction patterns indicating a similar preferred orientation of planes regardless of the kind of halogens such as F, Cl, Br, and I. The X-ray diffraction patterns on the cross-sectional planes being perpendicular to the growth direction of the crystals show that the growth direction of the crystals is mainly perpendicular to the *c*-axis whereas minor random orientation peaks of {h31}, {h11}, and {h01} planes are observed.

Typical infrared absorption spectra for the powder samples of In_4_Se_3−*x*_ (*x* = 0.65) and In_4_Se_3−*x*_*H*_0.03_ (*x* = 0.68; *H* = F, Cl, Br, I) crystals are shown in Figure 15b. All samples exhibit spectroscopically observable energy band gaps between 0.62 and 0.63 eV, which are consistent with a band gap between 0.5 and l.0 eV for In_4_Se_3_ with anisotropic band dispersions [19]. This indicates that the halogen substitution on the In_4_Se_3−*x*_ crystals does not influence on band gap widening despite of more electronegative character of halogen than selenium. Thus, the more ionic character in the In–H bond than the In–Se bond may result in the wider band gap in the In_4_Se_3−*x*_*H*_0.03_ crystal than the In_4_Se_−*x*_ crystal. However, a nominal concentration of halogen-substitution in this work is just ~1.3 mol% on anionic sites, which may be too small to result in band gap widening.

**Figure 16:**
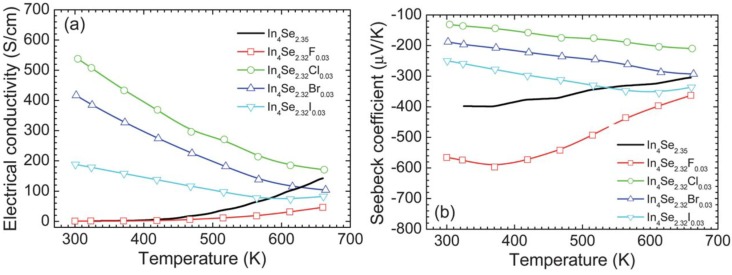
Temperature dependence of (**a**) electrical conductivity and (**b**) Seebeck coefficient of the crystals of In_4_Se_3−**x**_*H*_0.03_ (*x* = 0.68; H = F, Cl, Br, I) and In_4_Se_2.35_ [32].

The temperature dependent electrical conductivities of the crystals of In_4_Se_3−*x*_ (*x* = 0.65) and In_4_Se_3−*x*_*H*_0.03_ (*x* = 0.68; *H* = F, Cl, Br, I) are plotted in Figure 16a. The thermoelectric properties were measured along the *bc*-plane of the crystals because the electrical conduction is thought to be dominant along the plane [8] and the high thermoelectric performance was revealed along the plane [15]. Here, the electrical and thermal properties have been measured in the same direction.

The room temperature electrical conductivities of the crystals of In_4_Se_2.35_, In_4_Se_2.32_F_0.03_, In_4_Se_2.32_Cl_0.03_, In_4_Se_2.32_Br_0.03_, and In_4_Se_2.32_I_0.03_ are ~1.1, ~0.9, ~537.6, ~416.7, and ~188.3 S/cm, respectively. The chlorine-, bromine-, and iodine-substituted In_4_Se_3−*x*_*H*_0.03_ crystals exhibit significantly higher room temperature electrical conductivities than the un-substituted and fluorine-substituted crystals. For instance, the crystal of In_4_Se_2.32_Cl_0.03_ shows ~500 times higher room temperature electrical conductivity than the crystal of In_4_Se_2.35_. The electrical conductivity σ is expressed as σ *= ne*μ, where *n* is the carrier concentration, *e* the electrical charge, and μ the carrier mobility. The increase in σ should result from either (both) the increase in *n* or (and) the increase in μ. Carrier concentration of the crystals measured by Hall effect will be shown below in order to understand the origin of the increase in σ. The respective electrical conductivities at ~660 K of the crystals of In_4_Se_2.35_, In_4_Se_2.32_F_0.03_, In_4_Se_2.32_Cl_0.03_, In_4_Se_2.32_Br_0.03_, and In_4_Se_2.32_I_0.03_ are ~142.3, ~46.3, ~170.6, ~103.4, and ~82.6 S/cm. The chlorine-, bromine-, and iodine-substituted In_4_Se_3−*x*_*H*_0.03_ crystals show metallic (degenerate semiconducting) behaviors indicating that the electrical conductivity decreases with increasing temperature while the unsubstituted and fluorine substituted crystals display typical semiconducting behaviors.

Table 5 shows both the electron concentrations and the Hall mobilities of the crystals of In_4_Se_2.35_, In_4_Se_2.32_F_0.03_, In_4_Se_2.32_Cl_0.03_, In_4_Se_2.32_Br_0.03_, and In_4_Se_2.32_I_0.03_. The room temperature electron concentrations show that the lighter and smaller halogen-substituted crystals display a slightly higher electron concentration, which is probably due to the atomic size difference between selenium and the halogens such as Cl, Br, and I. For instance, the empirically measured atomic radii of Se, F, Cl, Br, and I are 1.15, 0.5, 1, 1.15, and 1.40 Å, respectively [33]. Compared to the atomic radius of selenium, the slightly smaller chlorine atom may be more easily occupied at the Se site in the lattice of the 4:3 indium selenide than the much smaller fluorine, the same size bromine, and the larger iodine atom. It has been reported that the crystal structure of In_4_Se_3_ consists of [(In_3_)^5+^(Se_3_)^6−^]^1−^ anionic layers (Se1, Se2, Se3 sites) and In^1+^ cations with a weak van der Waals interaction along the *a*-axis between the layers and strong covalent-ionic interaction in the *bc*-planes within the layer [17,34,35].

**Table 5:** Formation energies in eV at each selenium site for the compositions of In_4_Se_3−*x*_*H*_0.03_ (*x* = 0.06; *H* = F, Cl, Br, I) and room temperature carrier concentrations and Hall mobilities of the crystals of In_4_Se_3−*x*_*H*_0.03_ (*x* = 0.68; *H* = F, Cl, Br, I) and In_4_Se_2.35_ [32].

H	Se1	Se2	Se3	*n_H_* (×10^17^ cm^−3^)	μ *_H_* (cm^2^·V^−1^·s^−1^)
F	1.46	1.54	1.31	0.45	124.8
Cl	1.27	1.14	1.12	41	819.5
Br	1.25	1.12	1.16	35	744.1
I	1.25	1.11	1.22	29	405.8
In_4_Se_2.35_	–	–	–	41	1.7

The formation energies of the compositions of In_4_Se_3−*x*_*H*_0.03_ (*x* = 0.06; *H* = F, Cl, Br, I) at each Se sites (Se1, Se2, and Se3 site) were calculated within the density functional theory. The formation energies of the compositions of In_4_Se_3−*x*_H_0.03_ (*x* = 0.06; H = F, Cl, Br, I) at each Se site in Table 5 show that the halogen-substitution at the Se3 site is energetically most favorable for H = F and Cl while the Se2 site is most preferable for H = Br and I. Based on the formation energy calculation results, the degree of energetic preference at a certain selenium site is approximately similar among different halogens. However, it is notable that the fluorine-substituted crystal does have a much lower electron concentration of the order of 10^16^ cm^−3^ than other halogen-substituted ones, which is even lower than that of the unsubstituted crystal of In_4_Se_3−*x*_ (*x* = 0.65). We may speculate that the fluorine element, unlike other halogen elements, may be preferentially occupied either at the Se vacancy sites (V_Se_) or at the interstitial sites (I) in the lattice of In_4_Se_3−*x*_ (*x* = 0.65) rather than at the Se sites (Se) and thus the fluorine-substitution may decrease the electron concentration because of hole character of either the defect F_VSe_^1−^ (an atom of F located at the Se vacancy site) or the defect F_I_^1−^ (an atom of F located at the interstitial site) rather than electron character of the defect F_Se_^1+^ (an atom of F located at the Se sublattice). We do another calculation of formation energies in order to clarify why the F-substituted sample has much lower electron concentration compared to the rest of samples.

Table 6 shows that the interstitial occupation of F is more energetically stable than the lattice substitution, whereas the opposite occurs for Cl, Br, and I. Our speculation regarding lower electron concentration in the fluorine-substituted sample is in good agreement with the calculations of formation energies. The chlorine-, bromine-, and iodine-substituted In_4_Se_3−*x*_H_0.03_ crystals show electron concentrations comparable to the unsubstituted In_4_Se_3−*x*_ crystal, while the fluorine-substituted crystal exhibits an electron concentration nearly two orders lower than the unsubstituted crystal. The Hall mobility μ_H_ is expressed as μ_H_ = σ*/ne* = σ *R_H_*, where *R_H_* is the Hall coefficient of the crystals. Except for fluorine, it appears that the heavier halogen-substituted In_4_Se_3−*x*_*H*_0.03_ crystal shows a slightly lower Hall mobility because of stronger electron–phonon scattering for heavier halogen-substituted crystals, which is mainly due to the heavier atomic mass of the heavier halogen elements. It is quite notable that the halogen substituted In_4_Se_3−*x*_*H*_0.03_ crystals, even that with fluorine, exhibit significantly higher Hall mobilities than the unsubstituted crystal. For instance, the room temperature Hall mobility of the crystal of In_4_Se_2.32_Cl_0.03_ is ~500 times higher than the unsubstituted crystal of In_4_Se_2.35_. Thus, the substantial increase in electrical conductivity of the halogen-substituted In_4_Se_3−*x*_*H*_0.03_ crystals should result from a remarkable increase in Hall mobility.

**Table 6:** Formation energies in eV for the compositions of In_4_Se_2.94_H_0.06_ (substitution), In_4_Se_2.88_H_0.06_ (substitution + vacancy), and In_4_Se_3_H_0.06_ (interstitial) [32].

Halogen	Substitution	Substition + Vacancy	Interstitial
F	1.31	1.04	0.60
Cl	1.12	1.02	1.28
Br	1.12	0.98	1.37
I	1.11	0.97	1.42

Usually, one observes degradation of mobility upon substitution, which is mainly due to enhanced carrier scattering. However, the experimental observation of enhanced carrier mobility upon halogen-substitution is interesting and thus it is useful to further elucidate the reason of the enhanced mobility, at least some possible explanations. We may speculate that either the electron scattering may be weakened through the halogen-substitution for some reason, or that the charge scattering mechanism of the halogen-substituted In_4_Se_3−*x*_*H*_0.03_ crystals may be quite different compared to that of the unsubstituted In_4_Se_3−*x*_ crystal, thus resulting in a significant enhancement of the Hall mobility of the halogen-substituted crystals. A detailed analysis of the charge scattering mechanism would be desirable and thus we utilized a single parabolic band (SPB) model [36] to obtain room temperature carrier mobilities of the halogen-substituted In_4_Se_3−*x*_*H*_0.03_ crystalline samples. The SPB model is derived from solutions to the Boltzmann transport equation within the relaxation time approximation. The reduced chemical potential across measured temperature range is firstly estimated in the SPB model from temperature dependent Seebeck coefficient. The effective mass is secondly estimated from the experimental carrier concentration and reduced chemical potential. The carrier mobility is finally estimated from the effective mass and a constant relaxation time of 2.2 × 10^−14^ s which was used in reference [11] (see Equation (5) in reference [32]).

Table 7 shows calculated effective masses and carrier mobilities of the samples at the assumption of acoustic phonon scattering within the framework of Boltzmann transport equation. For example, the carrier mobility of 130 cm^2^·V^−1^·s^−1^ was estimated in the Cl-substituted crystalline sample with the assumption of acoustic phonon scattering while that of 819.5 cm^2^·V^−1^·s^−1^ was experimentally obtained. There is a large discrepancy between the experimental carrier mobility and the estimated carrier mobility. Therefore, we believe that either a combination of charge scatterings including acoustic phonon scattering, ionized impurity scattering, and neutral impurity scattering can govern the charge scattering mechanism of the halogen-substituted samples or the SPB model can fail because of multiple bands contributing in the 4:3 indium selenide like heavy and light hole band in PbTe or nonparabolicity present in almost all systems.

**Table 7:** Room temperature properties of In_4_Se_2.35_ and In_4_Se_3−*x*_H_0.03_ (*x* = 0.68; H = F, Cl, Br, I) crystalline samples, where the calculated m_cal_^*^ and *m_cal_* are obtained assuming acoustic phonon scattering within the framework of Boltzmann transport equation [32].

Composition	μ *_H_* (cm^2^·V^−1^·s^−1^)	μ *_cal_* (cm^2^·V^−1^·s^−1^)	m_cal_^*^ (*m_e_*)
In_4_Se_2.35_	1.7	19	1.75
In_4_Se_2.32_F_0.03_	124.8	98	0.35
In_4_Se_2.32_Cl_0.03_	819.5	130	0.19
In_4_Se_2.32_Br_0.03_	744.1	97	0.30
In_4_Se_2.32_I_0.03_	405.8	70	0.45

Figure 16b shows the Seebeck coefficient as a function of temperature for the crystals of In_4_Se_3−*x*_ (*x* = 0.65) and In_4_Se_3−*x*_H_0.03_ (*x* = 0.68; H = F, Cl, Br, I). The quasi-onedimensional In–Se chain in the CDW plane for the 4:3 indium selenide is a main factor contributing to the thermoelectric performance, which is because of the enhancement on the Seebeck coefficient due to the In–Se chain (quantum confinement) as well as the reduction on the lattice thermal conductivity due to the Peierls distortion in the CDW plane.

**Figure 17:**
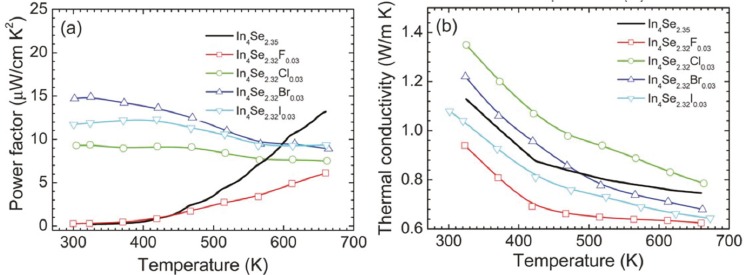
Temperature dependence of (**a**) power factor and (**b**) thermal conductivity of the crystals of In_4_Se_3−*x*_H_0.03_ (*x* = 0.68; H = F, Cl, Br, I) and In_4_Se_2.35_ [32].

The absolute values of Seebeck coefficient of the chlorine-, bromine-, and iodine-substituted In_4_Se_3−*x*_H_0.03_ crystals increase with increasing temperature, which is consistent with their electrical conductivity behaviors. It has been reported that the chlorine doping on In_4_Se_3−*x*_ only increased its chemical potential, giving rise to an increase in the electron concentration and thus the temperature dependent Seebeck coefficient of the chlorine doped crystal was able to be reproduced by the Boltzmann transport calculation with a fixed chemical potential [29]. This implies that the halogen-substitution on In_4_Se_3−*x*_ should not weaken the role of the In–Se chain on Seebeck coefficient compared to the halogen-free In_4_Se_3−*x*_ crystals. The corresponding temperature dependent power factors of the unsubstituted and halogen substituted crystals are plotted in Figure 17a.

It is quite notable that, except for fluorine, the room temperature power factors of the halogen-substituted crystals are significantly higher than that of the unsubstituted crystal, which is mainly due to the significant increase in the electrical conductivity. For instance, the bromine-substituted crystal shows a room temperature power factor which is approximately 80 times higher than the unsubstituted crystal. This indicates that the power factors of the chlorine-, bromine-, and iodine-substituted crystals slightly decrease with a rise in temperature while those of the unsubstituted and fluorine-substituted crystals rapidly increases with increasing temperature.

Figure 17b shows the temperature dependent total thermal conductivities of the crystals of In_4_Se_3−*x*_ (*x* = 0.65) and In_4_Se_3−*x*_*H*_0.03_ (*x* = 0.68; *H* = F, Cl, Br, I). The total thermal conductivities of all the crystals decrease with increasing temperature. The room temperature total thermal conductivities of the crystals ranges from 1.1 ~ 1.3 W·m^−1^·K^−1^, respectively. Thus, the room temperature lattice thermal conductivities are expected to be 0.9~1.1 W·m^−1^·K^−1^ when the Lorenz number is assumed to be 2.45 × 10^−8^ V^2^·K^−2^ for degenerate semiconductor. These are substantially low at room temperature, which is comparable to nanostructured LAST (Pb–Ag–Sb–Te) and Bi–Sb–Te samples [25,29]. Based on the expected room temperature lattice thermal conductivity data, the halogen-substitution may not have a negative influence on the effect of the Peierls distortion on the reduction in the lattice thermal conductivity.

**Figure 18:**
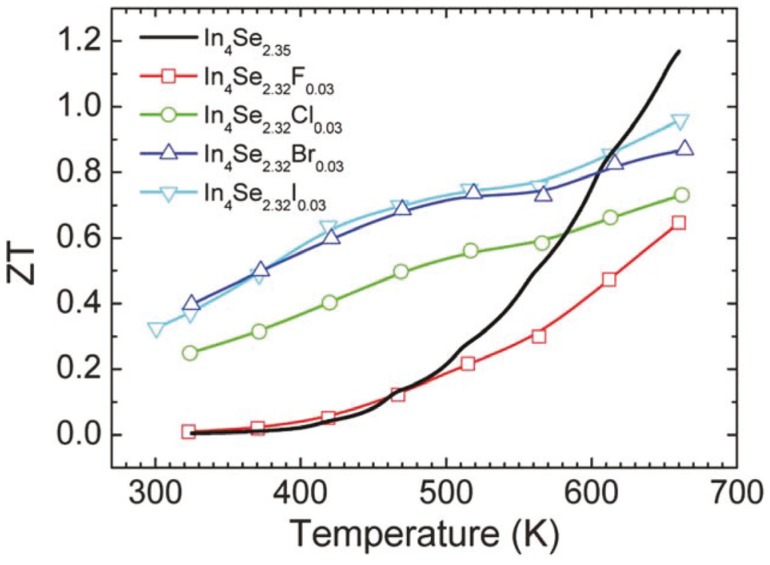
Temperature dependence of the thermoelectric figure of merit of the crystals of In_4_Se_3−*x*_H_0.03_ (*x* = 0.68; H = F, Cl, Br, I) and In_4_Se_2.35_ [32].

Figure 18 shows the dimensionless thermoelectric figure of merit *ZT* as a function of temperature for the unsubstituted and halogen-substituted crystals. The *ZT* increases with increasing temperature. It is quite notable that the room temperature *ZT* values of the Cl-, Br- and I-substituted crystals are substantially higher than that of the unsubstituted crystal. For instance, the room temperature *ZT* of the crystal of In_4_Se_2.32_Cl_0.03_ is about 40 times higher than that of the crystal of In_4_Se_2.35_. The respective *ZT* values at 660 K for the crystals of In_4_Se_2.35_, In_4_Se_2.32_F_0.03_, In_4_Se_2.32_Cl_0.03_, In_4_Se_2.32_Br_0.03_, and In_4_Se_2.32_I_0.03_ are 1.2, 0.6, 0.7, 0.9, and 1.0. Note that the crystal of In_4_Se_2.67_Cl [29] has been reported to exhibit a higher *ZT* than the crystal of In_4_Se_2.32_Cl_0.03_, and thus we grew the crystals of In_4_Se_2.67_H_0.03_ (H = F, Br, I) and measured their thermoelectric properties. However, the *ZT* values of the crystals of In_4_Se_3−*x*_H_0.03_ (H = F, Br, I) were unfortunately nearly as same as those of the crystals of In_4_Se_2.32_H_0.03_ (H = F, Br, I). Therefore, in this work we only focused on the improvement of thermoelectric performance in the crystals of In_4_Se_2.32_H_0.03_ (H = F, Cl, Br, I) compared to the crystal of In_4_Se_2.35_.

#### 2.3.3. Multiple Elements Doping and Selenium Deficiency in Polycrystalline In_4_Pb_0.01_Sn_0.03_Se_3−*x*_ Compounds

Recently, the high *ZT* = 1.4 at 733 K for Pb-/Sn-codoped In_4_Pb*_x_*Sn*_y_*Se_3_ polycrystalline compounds was reported [37]. The Boltzmann transport calculation of In_4_Se_3−δ_ shows that the high power factor *S^2^*σ can be expected for increasing chemical potential, in other words electron doping [15,29]. In our previous study, the cationic elements substitution was not effective due to the decrease of carrier mobility [27]. Lin *et al.* noticed that the Pb and Sn substitute at different In-sites (In4 and In2 sites respectively) when they re-examine the formation energy calculation of In_4_Se_3_ [27]. The crystal structure of In_4_Se_3_ consists of In^1+^ cations and [(In_3_)^5+^(Se_3_)^6−^]^1−^ anionic staking layers along the *a*-axis [35,38,39].

Despite non-systematic dependence on the Pb and Sn concentrations, it is believed that the co-doping is very effective for increasing *ZT* value of In_4_Se_3_ phase [37]. In the In_4_Se_3_ system, the Se-deficiency is very important for high thermoelectric performance. The non-stoichiometric Se-deficiency has an effect of hole localization with dispersive electron conduction resulting in the quasi-one-dimensional electronic transport [21]. From the formation energy calculation, the Se deficiency is in the [(In_3_)^5+^(Se_3_)^6−^]^1−^ anionic layer of the In_4_Se_3_ structure. The vacant site of a dangling bonded Se (Se3 site) is more stable than other Se sites. The theoretical calculation proved that the Se vacancy strongly suppresses phonon propagation along the plane of charge density wave [39]. In addition, the Se deficiency of the In_4_Se_3−*x*_ can decrease the electrical resistivity by increasing carrier concentration [21,40,41] as well as the Hall mobility (μ*_H_*) [21,42]. Therefore, we studied the Se deficiency effect on the Pb-/Sn-codoped In_4_Pb_0.01_Sn_0.03_Se_3−*x*_ polycrystalline compounds.

**Figure 19:**
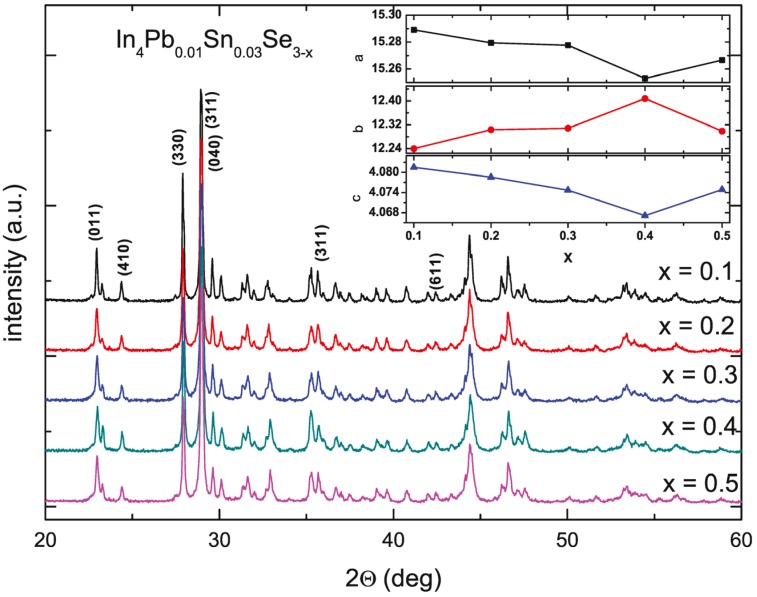
The powder X-ray diffraction patterns of In_4_Pb_0.01_Sn_0.03_Se_3−*x*_ (*x* = 0.1, 0.2, 0.3, 0.4, and 0.5) polycrystalline compounds. The inset shows the lattice parameters of the compounds. Reproduced with permission from Elsevier B.V., 2014 [43].

Figure 19 shows the powder XRD pattern of the annealed In_4_Pb_0.01_Sn_0.03_Se_3−*x*_ (*x* = 0.1, 0.2, 0.3, 0.4, and 0.5) polycrystalline compounds [43]. The patterns represent a single phase of In_4_Se_3_ with no distinguishable impurity phases. The inset of Figure 19 depicts the lattice parameters of In_4_Pb_0.01_Sn_0.03_Se_3−*x*_ polycrystalline compounds with different Se-deficiency. The lattice parameters of *a*- and *c*-axis are systemically decreased and *b*-axis is increased with increasing Se deficiency from *x* = 0.1 to 0.4 indicating systematic control of Se deficiency. For *x* = 0.5 compound, the lattice parameters are abruptly changed, indicating the solubility limit of Se-deficiency. From the crystal structure of In_4_Se_3_, the Se vacancy is energetically favorable at the dangling bonded Se3-site [19,37]. If the vacancy in the Se3-site is employed, the charge re-distribution can relax the cluster. Therefore we anticipate that the lattice is elongated along the *b*-axis while the lattices along the *a*- and *c*-axis are compressed due to the cluster distortion.

**Figure 20:**
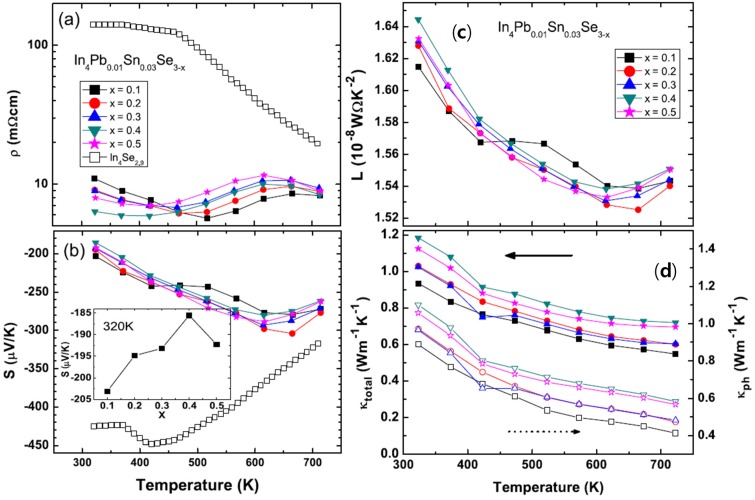
Temperature-dependent electrical resistivity ρ*(T)* (**a**), Seebeck coefficient *S(T)* (**b**), Temperature-dependent Lorenz number *L* (**c**), total thermal conductivity κ*_tot_* (closed symbols, left axis) and lattice thermal conductivity κ*_ph_* (open symbols, right axis) (**d**) of In_4_Pb_0.01_Sn_0.03_Se_3−*x*_ (*x* = 0.1, 0.2, 0.3, 0.4, and 0.5) polycrystalline compounds. Reproduced with permission from Elsevier B.V., 2014 [43].

The temperature dependent electrical resistivities ρ*(T)* of polycrystalline compounds In_4_Pb_0.01_Sn_0.03_Se_3−*x*_ (*x* = 0.1, 0.2, 0.3, 0.4, and 0.5) [43] and In_4_Se_2.9_ (open square for comparison [21]) are presented in Figure 20a. The ρ*(T)* of Pb-/Sn-codoped polycrystalline compound In_4_Pb_0.01_Sn_0.03_Se_2.9_ is significantly decreased than the one of In_4_Se_2.9_. The ρ*(T)* s near room temperature of Pb-/Sn-codoped and Se-deficient polycrystalline compounds are decreased with increasing Se-deficiency except for x = 0.5 case. The decrease of electrical resistivity is similar to those of In_4_Se_3−*x*_ polycrystalline compounds [21,40,41,44]. From the effective carrier concentration *n_H_* and Hall mobility μ*_H_*, calculated by Hall resistivity ρ*_xy_* measurement (Table 8), the carrier concentrations are increased with increasing Se-deficiency while electrical resistivity and Hall mobility of the compounds are decreased up to *x* = 0.4. The Pb-/Sn-codoping significantly increase the carrier density from 2.09 × 10^17^ cm^−3^ for polycrystalline compounds of In_4_Se_2.9_ [21] to 2.67 × 10^18^ cm^−3^ for In_4_Pb_0.01_Sn_0.03_Se_2.9_.

**Table 8:** The Hall carrier density $n_{H}$, electrical resistivity ρ, Hall mobility $\left|\mu_{H}\right|$, Seebeck coefficient *S* and effective mass of electron m^*^ of In_4_Pb_0.01_Sn_0.03_Se_3−*x*_ (*x* = 0.1, 0.2, 0.3, 0.4, and 0.5) polycrystalline compounds. Reproduced with permission from Elsevier B.V., 2014 [43].

x	$n_{H}$ (10^18^ cm^−3^)	ρ (mΩ·cm)	$\mu_{H}$ (cm^2^·V^−1^·S^−1^)	S (μV/K)	$m^{*}$ ($m_{e}$)
0.1	2.67	10.944	213.60	−203.14	0.182
0.2	4.16	9.056	165.67	−194.87	0.235
0.3	4.99	8.925	140.14	−193.20	0.263
0.4	6.26	6.332	157.46	−185.56	0.294
0.5	4.37	7.928	180.15	−192.32	0.240

There are broad humps of electrical resistivity near 600~650 K. The peak positions of ρ*(T)* are decreased with increasing Se-deficiency from 665 K (*x* = 0.1) to 615 K (*x* = 0.5). From the thermal measurements of thermogravimetric and differential thermal analysis (TG/DTA), we do not find any phase transformation at those temperatures. The broad increase of resistivity is observed at a charge density wave instability [7,45]. One possibility of the broad humps is caused by the charge density wave phase transitions [15]. However, it should be investigated as a further research to clarify the origin of abnormal increase of resistivity.

The temperature dependent Seebeck coefficients *S(T)* of In_4_Pb_0.01_Sn_0.03_Se_3−*x*_ (*x* = 0.1, 0.2, 0.3, 0.4, and 0.5) and In_4_Se_2.9_ polycrystalline compounds are shown in Figure 20b. Because the ρ*(T)*s are significantly decreased, the *S(T)* values are decreased for the Pb-/Sn-codoped polycrystalline compounds comparing with In_4_Se_2.9_. The maximum values of absolute Seebeck coefficients are appeared near the temperatures close to the temperature of broad shoulder in the electrical resistivity. The thermal band gap (*E_g_*) can be roughly estimated by the maximum Seebeck coefficient (*S*_max_) and the temperature of the *S*_max_, as following relation: *E_g_* = *2eS*_max_*T*_max_, where *e* is the electron charge [46]. The energy gaps are obtained ranging from 0.34 to 0.40 eV which are smaller than the one of In_4_Se_3_ (0.42 eV) [42]. The smaller thermal band gap of the compounds than the one of stoichiometric In_4_Se_3_ may come from the change of band structure by Pb-/Se-codoping and Se-deficiency. As shown in the inset of Figure 20a,b, the absolute Seebeck coefficients at room temperature follow the trade-off relationship with electrical resistivity.

Figure 20c shows the temperature dependent Lorenz number *L* [43]. The Lorenz numbers are very low compared with *L_0_* = 2.45 × 10^−8^ W·Ω·K^−2^. The low Lorenz numbers of the compounds may be affected by the change of Fermi energy [47]. From the obtained Lorenz number, we can calculate the lattice thermal conductivity κ*_ph_*. The total thermal conductivity κ (closed symbols, left axis) and lattice thermal conductivity κ*_ph_* (open symbols, right axis) are presented in Figure 20d. The κ and κ*_ph_* are increased with increasing Se-deficiency. It is consistent with the result of In_4_Se_3−*x*_ for high Se-deficiency (*x* > 0.05) [21]. The total thermal conductivity of In_4_Se_3−*x*_ (*x* > 0.05) is increased with increasing Se-deficient [21].

Basically, the low thermal conductivity of In_4_Se_3_ based compounds comes from the Peierls distortion due to quasi-onedimensional lattice and charge density wave instabilities [15]. Recent theoretical calculation of thermal conductivity by molecular dynamic simulation showed the Se-vacancy induces discontinuous charge density which causes the decrease of phonon transport [39]. In terms of this interpretation, the Pb- and Sn-codoped polycrystalline compounds might have a different charge density distribution. The employment of Se-vacancy can interact with Pb- and Sn-cation substitutions and has a role of the weakness of the decrease of phonon propagation, resulting in the increase of thermal conductivity by increasing Se-deficiency. The detailed phonon dispersion relation should be investigated in order to understand those phenomena.

**Figure 21:**
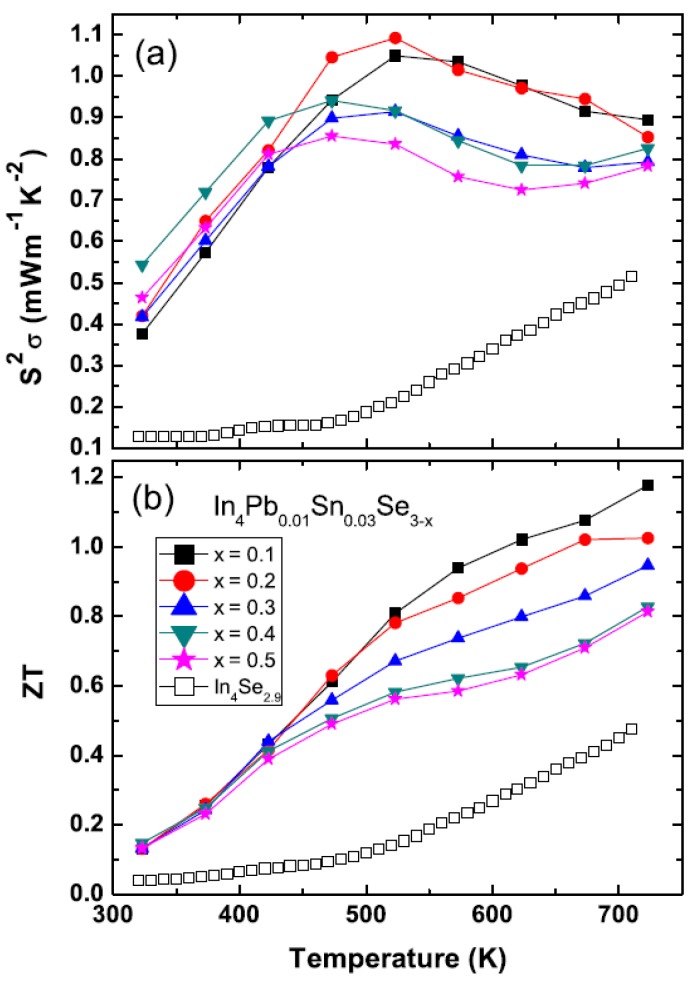
Temperature-dependent power factor *S*^2^σ (**a**), and dimensionless figure-of-merit *ZT* (**b**) of In_4_Pb_0.01_Sn_0.03_Se_3−_*_x_* (*x* = 0.1, 0.2, 0.3, 0.4, and 0.5) polycrystalline compounds. Reproduced with permission from Elsevier B.V., 2014 [43].

The temperature dependent power factors *S*^2^σ of polycrystalline In_4_Pb_0.01_Sn_0.03_Se_3−*x*_ (*x* < 0.5) and In_4_Se_2.9_ are presented in Figure 21a. The power factors of the Se-deficient and Pb-/Sn-codoped polycrystalline compounds (0.37–0.55 mW·m^−1^·K^−2^) have higher power factor than the ones of In_4_Pb_0.01_Sn*_y_*Se_3_ (0.1~0.19 mW·m^−1^·K^−2^) [29] near room temperature. Comparing with the In_4_Se_2.9_, the Pb-/Sn-codoped and Se-deficient compounds have significantly enhanced values of the power factor. The high power factor over a wide temperature range (450 K ≤ *T* ≤ 725 K) is very important ingredient for practical thermoelectric applications.

The thermoelectric figure-of-merit *ZT* of In_4_Pb_0.01_Sn_0.03_Se_3−*x*_ (*x* < 0.5) are increased with increasing temperature as shown in Figure 21b. The maximum *ZT* value reaches up to 1.2 at 723 K for polycrystalline In_4_Pb_0.01_Sn_0.03_Se_3−*x*_ (*x* = 0.1). Even though the *ZT* value of the *x* = 0.1 compound is lower than the one of In_4_Pb_0.01_Sn_0.03_Se_3_ (1.4 at 733 K) [37], if we define the operational temperature *T_op_* range as the temperature range for *ZT* > 1.0, the operational temperature range of *x* = 0.1 Se-deficient and Pb-/Sn-codoped polycrystalline compound (*T_op_* > 600 K) is a little bit increased rather than those of In_4_Pb_0.01_Sn_0.03_Se_3_ compound (*T_op_* > 620 K). In addition, the *ZT* values of In_4_Se_3−*x*_ significantly increased in the Pb-/Sn-codoped and Se-deficient polycrystalline compounds.

#### 2.3.4. Multiple Elements Doping with Chlorine Doping and Selenium Deficiency in In_4_Pb_0.01_Sn_0.03_Se_2.9_Cl*_x_* Polycrystalline Compounds

The halogen elements doping in the In_4_Se_3_ crystalline compounds In_4_Se_3−_*_x_*H*_y_* (H = F, Cl, Br, and I) is a good candidate to enhance chemical potential [32]. The Hall mobilities of the single crystalline In_4_Se_3−*x*_H_0.03_ are significantly increased by halogen doping. It was found that the Cl-doping is the most effective for increase of Hall mobility. The polycrystalline In_4_Pb_0.01_Sn_0.03_Se_2.9_ compounds showed maximum *ZT* value of 1.2 at 723 K [43]. However, the power factor of the In_4_Pb_0.01_Sn_0.03_Se_2.9_ polycrystalline compound can increase furthermore because it is still far from the optimized chemical potential 0.8 eV from the Boltzmann transport calculation [15,29]. The chemical potential of the In_4_Se_3_ phase can be more increased by electron doping. The Cl-doping is believed to be a good candidate to increase thermoelectric performance in the multiple elements-doped In_4_Pb_0.01_Sn_0.03_Se_2.9_Cl_x_ compounds.

**Figure 22:**
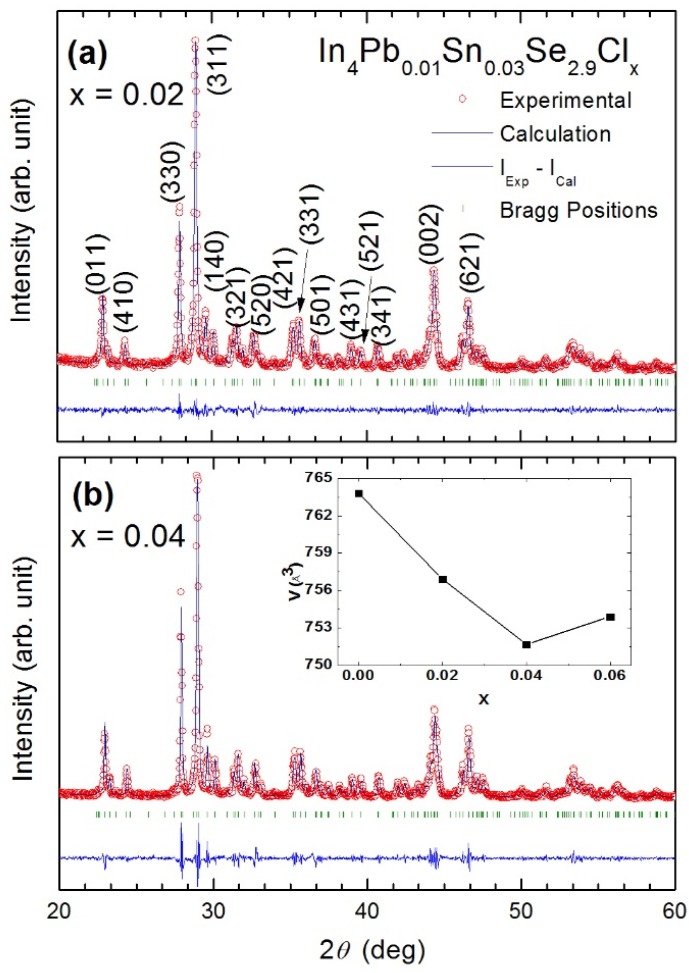
Powder X-ray diffraction profiles of In_4_Pb_0.01_Sn_0.03_Se_2.9_Cl*_x_* polycrystalline compounds (**a**) *x* = 0.02 and (**b**) *x* = 0.04. Reliability factors (R-factor) obtained from the fitting are as follows. *x* = 0.02: *R*_p_ = 6.75, *R*_wp_ = 8.78, *R*_exp_ = 7.30; *x* = 0.04: *R*_p_ = 9.10, *R*_wp_ = 11.3, *R*_exp_ = 7.34. Inset of (**b**) shows the lattice volume with respect to Cl concentration of the compounds [48].

Figure 22 shows Rietveld refinement analysis on powder X-ray diffraction (XRD) patterns of the prepared samples (*x* = 0.02 and *x* = 0.04) based on the orthorthombic *Pnnm* space group, respectively. The XRD patterns show a single phase of In_4_Se_3_ with no noticeable impurity peaks except *x* = 0.06. Because of impurity phase of InSe (Hexagonal, No. 194), we cannot analyze the XRD pattern by Rietveld refinement for the *x* = 0.06 compound. This indicates that *x* = 0.06 is the solubility limit of chlorine which induces the phase separation from In_4_Se_3_ to InSe phase. The lattice parameter is calculated as shown in Table 9. In the previous investigation of Se-deficient Pb/Sn co-doped polycrystalline compounds In_4_Pb_0.01_Sn_0.03_Se_3−*x*_ (*x* = 0.1, 0.2, 0.3, 0.4, and 0.5), the lattice parameters are systemically changed [43]. However, the lattice parameters of Cl-doped samples In_4_Pb_0.01_Sn_0.03_Se_2.9_Cl*_x_* do not show the systematic changes but show a decrease of lattice volume (inset of Figure 22) with increasing chlorine concentration up to *x* = 0.04 compound. Because the compound of *x* = 0.06 has InSe impurity phase, the lattice volume of the compound does not show systematic change.

**Table 9:** The lattice parameters of In_4_Pb_0.01_Sn_0.03_Se_2.9_Cl*_x_* (*x* = 0.0, 0.02, 0.04, and 0.06) polycrystalline compounds [48].

*x*	*a* (Å)	*b* (Å)	*c* (Å)	*V* (Å^3^)
0.00 [43]	15.29	12.24	4.08	764
0.02	15.19	12.29	4.06	757
0.04	15.20	12.19	4.06	752
0.06	15.22	12.20	4.06	754

From the formation energy calculation of halogen-substituted In_4_Se_3−*x*_H_0.03_ compound, the substitution and vacancy occupation at Se3-site by Cl-doping is the most stable state in In_4_Se_3−*x*_ [32]. The decrease of lattice volume by Cl-doping can be understood by the strong Coulomb interaction from the electronegativity of Cl and the Cl-substitution at Se3 site [49].

**Figure 23:**
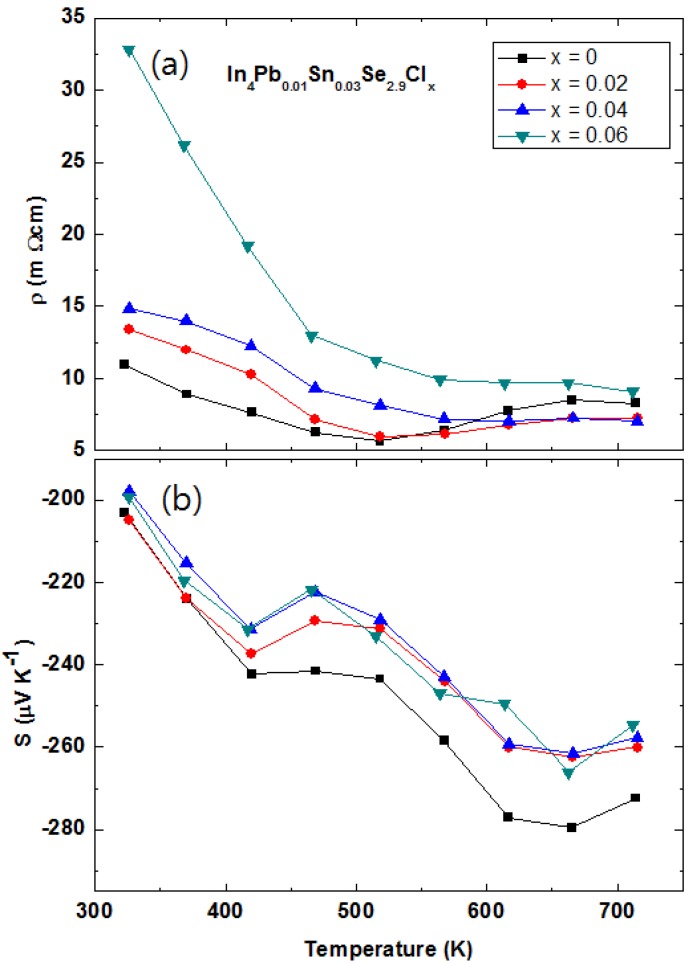
Temperature-dependent electrical resistivity ρ*(T)* (**a**) and Seebeck coefficient *S(T)* (**b**) of the In_4_Pb_0.01_Sn_0.03_Se_2.9_Cl*_x_* (*x* = 0.0, 0.02, 0.04, and 0.06) polycrystalline compounds [48].

The temperature dependent electrical resistivity of the polycrystalline In_4_Pb_0.01_Sn_0.03_Se_2.9_Cl*_x_* (*x* = 0.02, 0.04, and 0.06) polycrystalline compounds and *x* = 0.0 (Ref. [43]) are presented in Figure 23a. The electrical resistivity of Cl-doped compounds shows the semiconducting behavior in the measured temperature range. The increasing electrical resistivity with increasing Cl doping shows a similar behavior as In_4_Se_2.7_Cl*_x_* [31].

From the Hall resistivity ρ*_xy_* and electrical resistivity ρ measurements, we obtain the effective carrier concentration *n_H_* and Hall mobility μ*_H_*, which are listed in Table 10. The carrier concentration of the In_4_Pb_0.01_Sn_0.03_Se_2.9_Cl*_x_* (*x* = 0.02) compound shows the value of 1.58 times higher than the one of pristine In_4_Pb_0.01_Sn_0.03_Se_2.9_ polycrystalline compound. The carrier concentration does not increase with Cl doping for *x* = 0.04 and 0.06, implying the solubility limit of the compounds as in the case of the polycrystalline In_4_Se_2.7_Cl*_x_* polycrystalline compounds (*x* = 0~0.05) [31].

**Table 10:** The Hall carrier concentration *n_H_*, electrical resistivity ρ, Hall mobility μ*_H_*, negative Seebeck coefficient −|*S*|, and effective mass of carrier m* of the In_4_Pb_0.01_Sn_0.03_Se_2.9_Cl*_x_* (*x* = 0, 0.02, 0.04, and 0.06) polycrystalline compounds at 320 K [48].

*x*	*n_H_* (×10^18^ cm^−3^)	ρ (mΩ·cm)	μ_H_ (cm^2^·V^−1^·s^−1^)	−S (μV/K)	m* (*m_e_*)
0.00	2.67	10.9	214	203	0.18
0.02	4.22	13.4	111	205	0.25
0.04	4.09	14.9	103	198	0.24
0.06	3.99	32.9	48	199	0.23

The Hall mobilities of the In_4_Pb_0.01_Sn_0.03_Se_2.9_Cl*_x_* polycrystalline compounds are decreased with increasing Cl-doping. In contrast with the result of In_4_Se_3−*x*_Cl_0.03_ single crystal [29], the polycrystalline compounds of In_4_Se_2.7_Cl*_x_* [31] and In_4_Pb_0.01_Sn_0.03_Se_2.9_Cl*_x_* decreased the Hall mobility by increasing the Cl-doping. It implies that the Hall mobility of polycrystalline In_4_Se_3_ is decreased by the excess Cl-impurity scattering at the grain boundary. However, the CuBr-doped In_4_Se_2.5_ polycrystal shows the increased Hall mobility [50].

The increase of Hall mobility in bulk crystalline materials is caused by the enhancement of crystallinity in chlorine doped In_4_Se_3−*x*_Cl_0.03_ single crystal [29]. For example, the x-ray diffraction patterns for the cross-sectional planes of crystalline ingots show well aligned crystal orientation. However, for polycrystalline materials, the Hall mobility of chlorine doped samples is decreased with increasing chlorine doping concentration [29]. This implies that the chlorine itself acts as scattering center of carriers but it helps to enhance crystallinity during crystal growth. The doped chlorine can increase Hall carrier density, but excess chlorine can precipitate at the grain boundaries, resulting in the decrease of Hall mobility. On the other hand, the single crystalline materials have less effect on grain boundary, thereby the enhanced crystallinity can increase Hall mobility of carriers.

The temperature dependent Seebeck coefficient *S*(*T*) of the polycrystalline In_4_Pb_0.01_Sn_0.03_Se_2.9_Cl*_x_* (*x* = 0.02, 0.04, and 0.06) compounds are decreased with Cl-doping as compared with *x* = 0.0 as shown Figure 23b. From the Hall carrier concentration and the measured Seebeck coefficient, the calculated effective masses of the carriers are listed in Table 10. The effective mass of carrier of *x* = 0.02 sample is obviously increased as compared the one of *x* = 0.0, even though the Cl-doping dependence is not clear. The increased effective mass of carrier indicates less dispersive electron band than the non-Cl-doped sample. From the effective thermal band gap relation, *E_g_ = 2eS*_max_*T*_max_ [46], we can estimate the band gap as 0.372 eV (*x* = 0.0) [40] and 0.347 eV (*x* = 0.04). The Cl-doping on the In_4_Pb_0.01_Sn_0.03_Se_2.9_Cl*_x_* compounds induces the less dispersive and decreased band gap than those of the In_4_Pb_0.01_Sn_0.03_Se_2.9_ compound.

**Figure 24:**
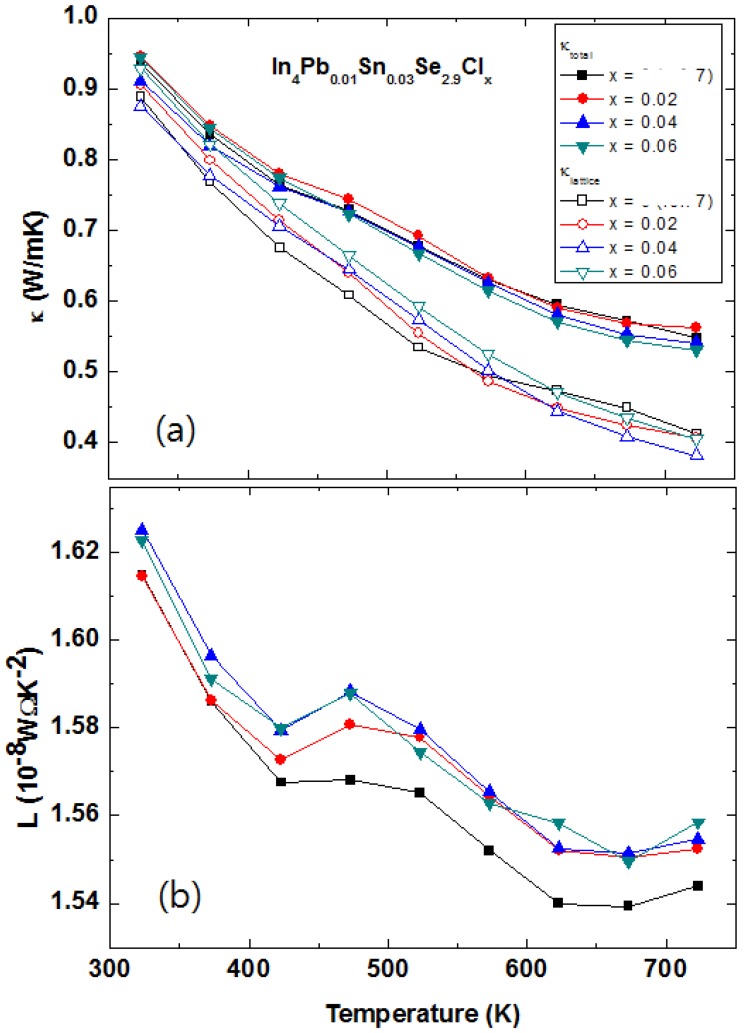
Temperature-dependent total thermal conductivity κ*_tot_* (closed symbols), lattice thermal conductivity κ*_ph_* (**a**), and Lorenz number *L*(*T*) (**b**) of the In_4_Pb_0.01_Sn_0.03_Se_2.9_Cl*_x_* (*x* = 0.0, 0.02, 0.04, and 0.06) polycrystalline compounds [48].

The total thermal conductivities of the polycrystalline In_4_Pb_0.01_Sn_0.03_Se_2.9_Cl*_x_* (*x* = 0, 0.02, 0.04 and 0.06) polycrystalline compounds are presented in Figure 24a. The thermal conductivities of (*x* = 0.0, 0.02, 0.04, and 0.06) are decreased with increasing temperature. This conventional 1/*T* behavior is mainly caused by an acoustic phonon contribution for the thermal transport. There is no systematic change of the κ*(T)* with respect to Cl-doping concentration. In general, the total thermal conductivity κ is composed of electrical κ*_e__l_* and lattice thermal conductivity κ*_ph_*. The electronic thermal conductivity κ*_el_* can be calculated by the Wiedemann-Franz law κ*_el_* = *L*_0_σ*T*, where *L*_0_, σ, and *T* are the Lorenz number, electrical conductivity, and absolute temperature, respectively. In usual cases, the Lorenz number is written as:

(5) $$L_{0}=\frac{\pi^{2}}{3}{\left(\frac{k_{B}}{e}\right)}^{2}=2.45\times 10^{-8}\;W\cdot \Omega \cdot K^{-2}$$

However, the Lorenz number is incorrect in correlated metal and many degenerated semiconductors. In order to get a more reliable Lorenz number, we calculated the Lorenz number by using the following equation:

(6) $$L={\left(\frac{k_{B}}{e}\right)}^{2}\left(\frac{\left(r+\frac{7}{2})F_{r+\frac{5}{2}}(\text{η}\right)}{\left(r+\frac{3}{2})F_{r+\frac{1}{2}}(\text{η}\right)}-{\left[\frac{\left(r+\frac{5}{2})F_{r+\frac{3}{2}}(\text{η}\right)}{\left(r+\frac{3}{2})F_{r+\frac{1}{2}}(\text{η}\right)}\right]}^{2}\right)$$

where *r* is the scattering parameter, η = *E_F_/k_B_T* is the reduced Fermi energy, and *F_n_(*η*)* is the *n*-th order Fermi integral given by:

(7) $$F_{n}\left(\text{η}\right)=\int_{0}^{\infty }\frac{x^{n}}{1+e^{x-\text{η}}}\text{d}x$$

For most cases, the scattering parameter for acoustic phonon scattering is *r* = −1/2. When we fit the measured Seebeck coefficient to the following equation with a free parameter η, we can get the Fermi integral η:

(8) $$S=\pm \frac{k_{B}}{e}\left\{\frac{\left(r+\frac{5}{2})F_{r+\frac{3}{2}}(\text{η}\right)}{\left(r+\frac{3}{2})F_{r+\frac{1}{2}}(\text{η}\right)}-\text{η}\right\}$$

By using the Fermi integral, the calculated temperature dependent Lorenz numbers of In_4_Pb_0.01_Sn_0.03_Se_2.9_Cl*_x_* (*x* = 0.0, 0.02, 0.04, and 0.06) are shown in Figure 24b. The calculated temperature dependent Lorenz number is lower than the conventional Lorenz number *L_0_* = 2.45 × 10^−8^ W·Ω·K^−2^. The low Lorenz numbers indicate that the electrical contribution for thermal transport is lower than conventional metals. Using the calculated Lorenz number and electrical resistivity, we can obtain the lattice thermal conductivity by subtracting the electrical thermal conductivity from the total thermal conductivity as shown Figure 24a (open symbols). The change of lattice thermal conductivity with respect to chlorine doping concentration of In_4_Pb_0.01_Sn_0.03_Se_2.9_Cl*_x_* (*x* = 0.0, 0.02, 0.04, and 0.06) polycrystals is not obvious. The similar thermal conductivity can be explained by the similarity of crystal structure and phonon dispersion relation between In_4_Se_3_ and InSe [51].

**Figure 25:**
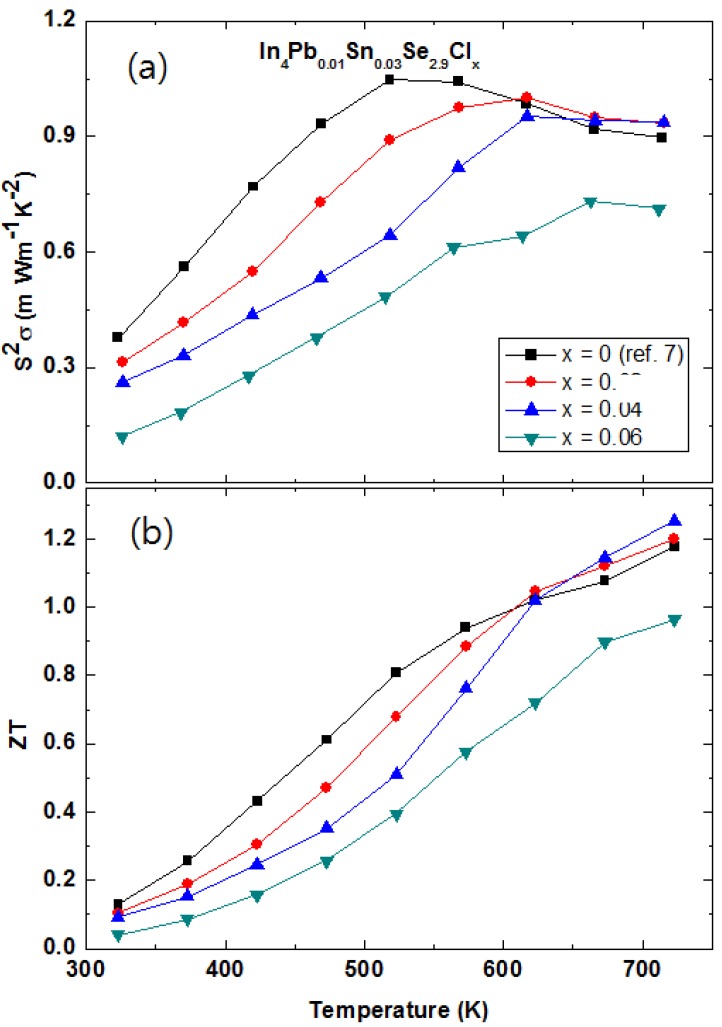
Temperature-dependent power factor *S*^2^σ (**a**) and *ZT* values (**b**) of the In_4_Pb_0.01_Sn_0.03_Se_2.9_Cl*_x_* (*x* = 0.0, 0.02, 0.04 and 0.06) polycrystalline compounds [48].

Figure 25a shows the temperature dependent power factor *S*^2^σ of the polycrystalline In_4_Pb_0.01_Sn_0.03_Se_2.9_Cl*_x_* (*x* = 0.0, 0.02, 0.04, and 0.06) compounds. The decreased power factor with increasing Cl-doping is mainly caused by the decreased Hall mobility near room- and mid-temperature range. On the other hand, the slightly increased power factors of *x* = 0.02 and 0.04 are obtained near 723 K. The compound of *x* = 0.06 shows a decreased power factor than the other samples (*x* = 0.0, 0.02, and 0.04) implying that the InSe phase separation is not good for increasing power factor in the In_4_Se_3_ phase.

The *ZT* values of the polycrystalline In_4_Pb_0.01_Sn_0.03_Se_2.9_Cl*_x_* (*x* = 0.0, 0.02, 0.04, and 0.06) compounds are presented in Figure 25b. The maximum *ZT* of *x* = 0.04 reaches up to 1.25 at 723 K, which is slightly increased value as compared with the one of pristine compound *x* = 0.0 (*ZT* = 1.2). The high *ZT* value over a wide temperature range in bulk crystals In_4_Se_3−*x*_Cl_0.03_ (1.53) is caused by the significant increase of Hall mobility, which is attributed from the enhancement of crystallinity by chlorine doping [29]. Because the polycrystalline In_4_Se_2.7_Cl*_x_* samples exhibit decreased *ZT* (0.67) value with increasing chlorine doping concentration, we believe that further enhancement of *ZT* can be possible in the compounds of In_4_Pb_0.01_Sn_0.03_Se_2.9_Cl*_x_* for single crystalline form. Therefore, we should investigate the thermoelectric properties of the single crystalline compounds in further research projects.

## 3. Experimental Section

### 3.1. Bulk Crystal Growth of In_4_Se_3−δ_ and In_4_Se_3−x_H_0.03_ (x = 0.68; H = F, Cl, Br, I)

Bulk crystal ingots of In_4_Se_3−δ_ and In_4_Se_3−*x*_H_0.03_ (*x =* 0.68; H = F, Cl, Br, I) were prepared by the Bridgman crystal growth method. Stoichiometric amounts of In, Se, and/or InH_3_ (H = F, Cl, Br, I) were loaded into a quartz tube under an argon atmosphere in order to keep the indium halides dry. The quartz tubes were vacuum sealed and heat treated at 860 K for 12 h in a rocking furnace for homogenization of the molten liquid. After the heat treatment of the compounds, the quartz tubes were loaded into a vertical Bridgman furnace. The compounds were melted at 860 K for 24 h and slowly pulled down at a constant rate of 2–5 mm/h.

### 3.2. Polycrystlline Compounds Preparation

The polycrystalline compounds were synthesized by solid state reaction at 550 °C for 24 h and spark plasma sintering under uniaxial pressure of 70 MPa and 420 °C for 5 min. For cation doped materials, the samples were synthesized by melting, quenching, and annealing process. The elements of In, Pb, Sn, and Se, InCl_2_, and so on were sealed in evacuated quartz tubes under high vacuum with respect to stoichiometric molar ratios. The quartz ampoules were heated at 1123 K for 24 h with following water quenching. The ingots were pulverized and sintered under argon atmosphere by hot-press at about 730 K for 1 h under uniaxial pressure of 70 MPa. In order to increase thermal stability, the pellets were annealed at 753 K for 5 days. The relative densities of annealed samples are above 95% (5.82~5.93 g/cm^3^) of the theoretical density.

### 3.3. Sample Characterizations

The chemical inhomogeneity was examined by the inductively coupled plasma spectroscopy (ICP) and electron dispersive spectroscopy (EDS) measurements. The variations of chemical concentration of three different slices of one crystal ingot from top to bottom were about ±2 at.%. The EDS measurements at several different points of a slice of the sample showed the chemical homogeneity within an experimental error. The crystal structure and phase of annealed samples are identified by the powder X-ray diffraction (XRD) using Cu kα radiation (D8 advance, Bruker, Billerica, MA, USA). The Rietveld refinement was carried out using FullProf software.

### 3.4. Thermoelectric Properties Measurements

We prepared disk-shaped and bar-type samples to measure the thermal conductivity κ and electrical transport properties (*S* and ρ), respectively. For anisotropic measurements, we cut the samples for two directions: along the growth direction and perpendicular to the growth direction. Typical sample sizes for thermal conductivity and electrical transport properties (*S* and ρ) measurements are of 10 mm diameter with 2 mm thickness and 10 mm long with (2~5) × (2~5) mm^2^ cross-sectional area, respectively.

High temperature thermal conductivity κ was obtained by the measurements of sample density ρ*_s_*, thermal diffusivity λ (by the laser flash method), and heat capacity *C_p_* (ULVAC, Chigasaki, Japan); κ *=* ρ*_s_*λ*C_p_*, where heat capacity *C_p_* was used the results from the Dulong-Petit fitting at high temperatures (*T* ≥ 300 K). The high temperature electrical resistivity ρ and Seebeck coefficient *S* were measured by the four-probe method (ZEM-2, ULVAC). The orthorhombic bar was placed in the hot and cold side plate and the voltage leads were contacted at the sample surface. By applying the heat and electric pulse, the Seebeck coefficient *S =* Δ*V*/Δ*T* and electrical resistivity ρ *=* Δ*V*/Δ*I* were measured simultaneously. The Hall resistivity ρ*_xy_* measurement was carried out by the five-contact AC (alternating current)-transport technique by the physical property measurement system (Quantum Design, San Diego, CA, USA). The Hall carrier concentration was calculated by the one-band model as following relation: *n_H_ =* −1/(*R_H_e*), where Hall coefficient *R_H_* = ρ*_xy_*/*H* and *e* = 1.602 × 10^−19^ C.

### 3.5. Band Structure Calculations

The first-principles calculation was performed by the pseudopotential plane wave method using the Vienna Ab initio Simulation Package (VASP). We adopted the generalized gradient approximation (GGA) implemented by Perdew, Burke, and Ernzerhof (PBE) for the exchange correlation energy functional with the spin-orbit interaction. The 8 × 8 × 24 Monkhorst and Pack scheme of *k*-point sampling is used for integration over the first Brillouin zone. The energy cutoff is chosen to be 240 eV and atomic positions are fully relaxed until all force components are smaller than 0.02 eV/Å. The unit cell is composed of 28 atoms with seven different sites (four sites for indiums and three sites for seleniums) and each site occupies four equivalent atomic positions. The calculated lattice parameters are of *a* = 15.429 Å, *b* = 12.442 Å and *c* = 34.142 Å, respectively, which are similar to the experimental values. For Se-deficient In_4_Se_3−δ_ (δ = 0.25) crystal, single Se atom was eliminated from the twelve Se atoms that occupied three crystallographically nonequivalent sites in the unit cell. We found that the configuration with vacant Se1 site is lower in energy than other configurations with Se2 and Se3 sites by 0.14 eV and 0.19 eV per unit cell, respectively.

The thermoelectric properties are calculated by using BoltzTraP program. Dense mesh of 19200 *k*-points in full Brilloun zone is used for the calculation. Exchange correlation energy functional is calculated using Engel-Vosko (EV) GGA for the Boltzmann transport calculation. Although PBE-GGA has been widely used for the self-consistent charge consistent calculation, it is known to underestimate the band gap. For a calculation of thermoelectric properties, a correct estimation of band gap is important. The EV-GGA has shown good agreement with experimental band gap. Indeed, PBE-GGA gives a band gap of 0.17 eV, but the EV-GGA gives a band gap of 0.57 eV, which is similar to the experimental value of 0.64 eV. We have used a rigid band approximation in the doping effect. In order to obtain the temperature dependent transport properties, we fix the chemical potential at μ = 0.22 eV, and use a constant time relaxation parameter τ = 2.2 × 10^−14^ s.

## 4. Conclusions

The Peierls lattice distortion can be regarded as a new pathway to enhance thermoelectric performance. The inherent nature of quasi-one-dimensional charge transport with strong electron-phonon coupling breaks translational symmetry of crystalline lattices. The low dimensional electronic and thermal properties of Peierls system result in the intrinsic nanowire-like transport of electrons and phonons. The electronic transport with reduced dimensionality can give rise to high Seebeck coefficients if we control the electron-hole band asymmetry. The phonon softening driven by the strong electron-phonon interaction lowers phonon energy and the lattice distortion along the conducting plane significantly increases the phonon scattering in charge density wave system. The increased phonon scattering with lowered phonon energy has the effect of reducing thermal conductivity. Based on this concept, high *ZT* on In_4_Se_3−δ_ bulk crystals along the crystalline plane with the charge density wave were reported.

The band structure of In_4_Se_3−δ_ shows anisotropic transport of carriers such as the significant electronic band dispersion along the *c*-axis, localized hole band along the *b*-axis, and the van der Waals energy gap along the *a*-axis. From the increase of the chemical potential by chlorine doping, the bulk crystalline In_4_Se_3−δ_Cl_0.03_ exhibited exceptionally high *ZT* over a wide temperature range. The thermoelectric properties of In_4_Se_3−δ_Cl_0.03_ are not optimized yet because the chemical potential is far from that of the highest power factor. If we increase chemical potential, the *ZT* value can be increased further in the In_4_Se_3−δ_ based materials. The Peierls distortion can be manifested by band structure engineering on the quasi-one-dimensional chain structure and two-dimensional layered structure materials.

In order to utilize waste heat power generation for Indium Selenides, high thermoelectric performance in polycrystalline compounds are highly desirable. The electrical resistivity ρ(*T*) and Seebeck coefficient *S*(*T*) of polycrystalline compounds of In_4_Pb_0.01_Sn_0.03_Se_3−*x*_ (*x* = 0.1, 0.2, 0.3, 0.4, and 0.5) are decreased with increasing Se deficiency by increasing carrier concentration. The room temperature power factors are increased by introducing Se-deficiency for Pb-/Sn-codoped compound. The maximum *ZT* value is reached up to 1.2 (at 723 K) for In_4_Pb_0.01_Sn_0.03_Se_3−*x*_ (*x* = 0.1) polycrystalline compound due to high Hall mobility 213.60 cm^2^·V^−1^·s^−1^ and increased carrier concentration.

The Cl-doped polycrystalline In_4_Pb_0.01_Sn_0.03_Se_2.9_Cl*_x_* (*x* = 0.0, 0.02, 0.04, and 0.06) compounds are synthesized well by the melting and solid state reaction with systemic change of lattice volume. Because of the chlorine impurity scattering, the electrical resistivities of the Cl-doped polycrystalline samples (*x* = 0.02, 0.04, 0.06) are increased as compared with non-Cl-doped sample (*x* = 0.0) near room temperature which is mainly caused by the decrease of Hall mobility by chlorine doping. However, the electrical resistivities of *x* = 0.02 and 0.04 are decreased at high temperature accompanying with smearing out of broad shoulder near 670 K. Consequently, it exhibits relatively low thermal conductivity and high power factor over a wide temperature range. The power factor and *ZT* values of the Cl-doped polycrystalline compounds (*x* = 0.02 and 0.04) were increased as compared with the non Cl-doped compound (*x* = 0.0) near 700 K. The maximum *ZT* value of 1.25 is relatively high *ZT* for polycrystalline In_4_Se_3_ based compounds. Further enhancement of thermoelectric performance can be expected in single crystalline compounds by promoting crystallinity of the samples as well as enhancement by multiple elements doping in polycrystalline compounds.
